# Enhancing osteogenic differentiation of diabetic tendon stem/progenitor cells through hyperoxia: Unveiling ROS/HIF‐1α signalling axis

**DOI:** 10.1111/jcmm.70127

**Published:** 2024-10-28

**Authors:** Ming Zhang, Guan‐Chun Dai, Yuan‐Wei Zhang, Pan‐Pan Lu, Hao Wang, Ying‐Juan Li, Yun‐Feng Rui

**Affiliations:** ^1^ Department of Orthopedics, Zhongda Hospital, School of Medicine Southeast University Nanjing People's Republic of China; ^2^ School of Medicine Southeast University Nanjing People's Republic of China; ^3^ Orthopaedic Trauma Institute (OTI) Southeast University Nanjing China; ^4^ Trauma Center, Zhongda Hospital, School of Medicine Southeast University Nanjing People's Republic of China; ^5^ Department of Geriatrics, Zhongda Hospital, School of Medicine Southeast University Nanjing People's Republic of China

**Keywords:** diabetic calcified tendinopathy, Hyperoxia, N‐acetyl‐L‐cysteine, osteogenic differentiation, reactive oxygen species, tendon stem/progenitor cells

## Abstract

Diabetic calcific tendinopathy is the leading cause of chronic pain, mobility restriction, and tendon rupture in patients with diabetes. Tendon stem/progenitor cells (TSPCs) have been implicated in the development of diabetic calcified tendinopathy, but the molecular mechanisms remain unclear. This study found that diabetic tendons have a hyperoxic environment, characterized by increased oxygen delivery channels and carriers. In hyperoxic environment, TSPCs showed enhanced osteogenic differentiation and increased levels of reactive oxygen species (ROS). Additionally, hypoxia‐inducible factor‐1a (HIF‐1a), a protein involved in regulating cellular responses to hyperoxia, was decreased in TSPCs by the ubiquitin‐proteasome system. By intervening with antioxidant N‐acetyl‐L‐cysteine (NAC) and overexpressing HIF‐1a, we discovered that blocking the ROS/HIF‐1a signalling axis significantly inhibited the osteogenic differentiation ability of TSPCs. Animal experiments further confirmed that hyperoxic environment could cause calcification in the Achilles tendon tissue of rats, while NAC intervention prevented calcification. These findings demonstrate that hyperoxia in diabetic tendons promotes osteogenic differentiation of TSPCs through the ROS/HIF‐1a signalling axis. This study provides a new theoretical basis and research target for preventing and treating diabetic calcified tendinopathy.

## 1 INTRODUCTION

Diabetic tendinopathy is a common disease of the musculoskeletal system in patients with diabetes mellitus (DM)^1^, ^2^ that causes chronic pain, movement restriction, tendon micro‐damage and rupture, and seriously affects patients' quality of life.^3^, ^4^ Calcific tendinopathy is a serious type of diabetic tendinopathy^5^, ^6^ mainly caused by calcium deposition in tendons, aggravating the clinical manifestations of diabetic tendinopathy.^7^ Currently, effective means of clinical prevention and treatment of calcific tendinopathy are limited because the pathogenesis remains unclear. Previous studies have shown that calcific tendinopathy is a complex process and its pathogenesis mainly includes the following hypotheses: (1) Degenerative calcification caused by repeated injury or vascular ischemia; (2) Reactive calcification mediated by active cells; (3) Ossification of fibrocartilage in tendon sheath; (4) Osteochondral metaplasia caused by mis‐differentiation of tendon stem/progenitor cells (TSPCs).^7^ In recent years, the mis‐differentiation of TSPCs have gradually become a research hotspot.^8^, ^9^, ^10^

It has been found that TSPCs may play an important role in the occurrence and development of calcific tendinopathy.^6^, ^11^ We originally proposed and continue to pay attention to changes in TSPCs differentiation function in tendon tissues in the pathogenesis of chronic tendinopathy.^10^, ^12^ Further, high‐sugar and advanced glycation end products promote the osteogenic differentiation ability of TSPCs in rats, leading to tendon calcification.^13^, ^14^ These studies demonstrate that TSPCs are important target cells in calcifying tendinopathy, and we hypothesize that TSPCs may also play an important role in diabetic calcified tendinopathy. Nevertheless, the specific regulatory mechanisms underlying increased osteogenic differentiation of diabetic TSPCs remain unclear.

Compared with that of non‐diabetic patients, the vascular cross‐section in tendon tissues of diabetic patients is larger, and the expression of vascular active factor, vascular endothelial growth factor (VEGF), is significantly increased.^15^ Additionally, compared with that of healthy rats, the blood vessels in the Achilles tendons of diabetic rats were also significantly increased and thickened, with a highly expressed VEGF.^16^ These studies revealed the expression of vascular and related active substances in tendon tissues of diabetic patients and rats, but the relationship between vascular and oxygen concentration was not clearly clarified. As everyone knows, blood vessels and red blood cells are the important channels and carriers of oxygen transport in organisms. The number and diameter of blood vessels and the number of red blood cells can directly affect the level of intravascular oxygen uptake, reflecting the oxygen content and concentration in the extracellular tissue environment.^17^, ^18^, ^19^ In this study, we will explain the positive relationship between blood vessels and tissue oxygen concentration, and verify that there may be a local hyperoxic environment in diabetes tendons. Additionally, hyperoxia can promote osteogenic cell differentiation and tissue calcification.^20^, ^21^, ^22^ But, the effect of hyperoxia on TSPCs differentiation has not been studied. So, on that basis, we speculate that hyperoxia may exist in diabetic tendon tissues and may be an important regulatory factor in promoting the osteogenic differentiation of TSPCs. However, the molecular mechanism by which hyperoxia promotes osteogenic differentiation of TSPCs in diabetic tendon is still unclear.

**FIGURE 1:**
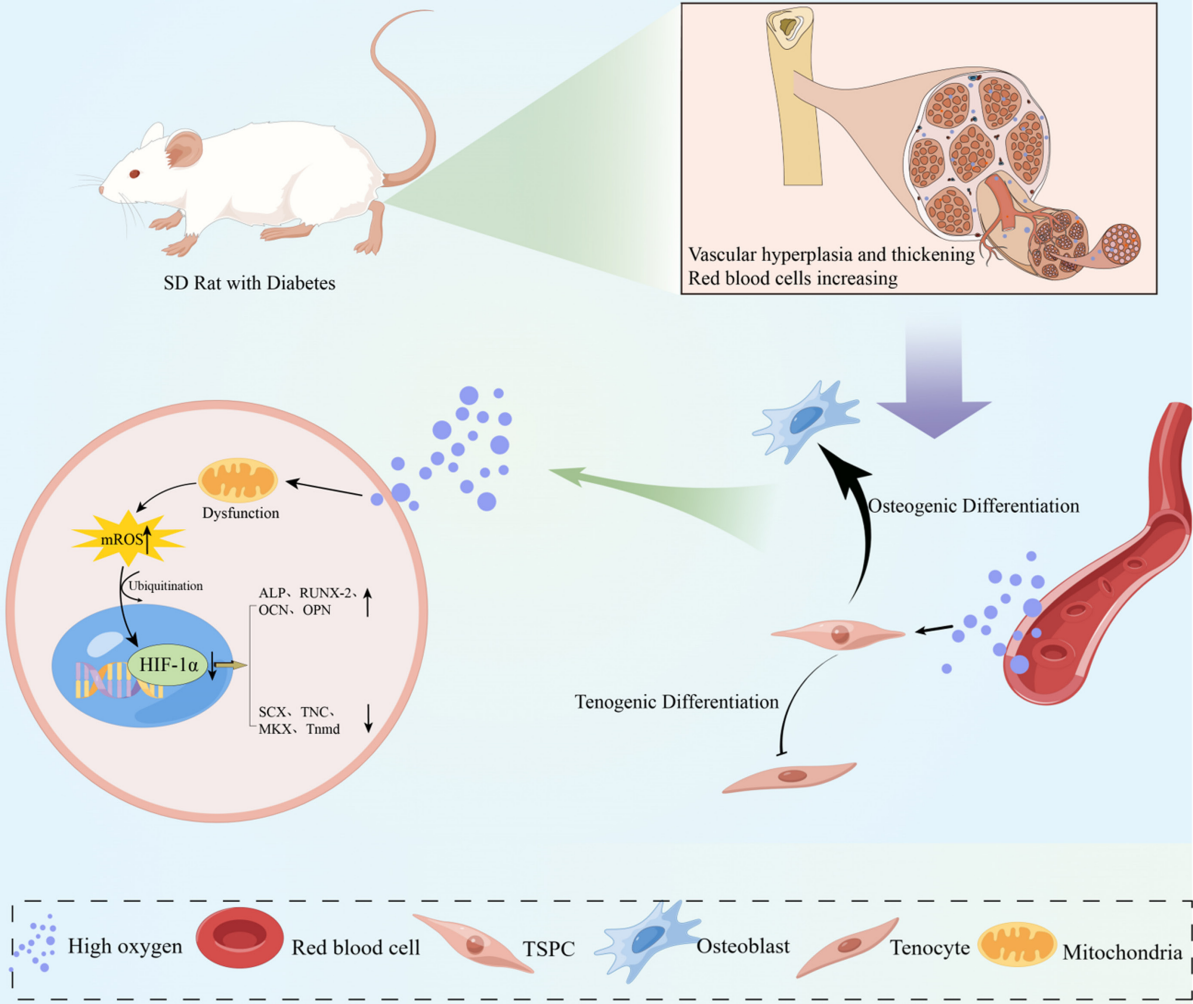
Molecular mechanism of ROS/HIF‐1a signalling axis mediated osteogenic differentiation of TSPCs in diabetic calcific tendinopathy. (By Figuredraw). TSPCs, Tendon stem/progenitor cells.

Hyperoxia increases reactive oxygen species (ROS) levels and enhances oxidative stress response in stem cells.^23^, ^24^ Mitochondria are the main sites of ROS generation and excessive ROS destroys the balance of oxidation and antioxidant effects in the cell, leading to oxidative stress, damage to cell and gene structure, and various diseases.^25^, ^26^ ROS is closely associated with the osteogenic differentiation ability of stem cells.^27^, ^28^ Hypoxia‐inducible factor‐1a (HIF‐1a) is a transcriptionally active nuclear protein involved in various biological processes.^29^, ^30^ HIF‐1a and its induced‐expression genes are critical for osteogenic cell differentiation and tissue calcification.^31^, ^32^ Additionally, ROS and HIF‐1a have a certain regulatory relationship, forming the ROS/HIF‐1a signalling axis and playing an important role in various cellular processes.^33^, ^34^, ^35^ These results suggest that ROS and HIF‐1a play an important role in osteogenic differentiation. However, the role of ROS/HIF‐1a signalling axis in osteogenic differentiation of TSPCs has not been investigated. Therefore, we speculate that the ROS/HIF‐1a signalling axis may play an important regulatory role in the osteogenic differentiation of TSPCs. Hence, our study aimed to explore the role of hyperoxia in the osteogenic differentiation of diabetic TSPCs and verify the molecular mechanism of the ROS/HIF‐1a signalling axis in the process of osteogenic differentiation (Figure 1), which might provide a novel research perspective and target for the clinical prevention and treatment of calcific tendinopathy with important application prospects.

## 2 MATERIALS AND METHODS

### 2.1 Reagent

Fetal bovine serum (FBS), penicillin/streptomycin, Dulbecco's modified Eagle's medium (DMEM), phosphate‐buffered saline (PBS) and 0.25% trypsin were purchased from Gibco BRL (NY, USA). Collagenase I, CoCl_2_, hydrogen peroxide (H_2_O_2_), N‐Acetyl‐L‐cysteine (NAC) and MG132 were also purchased from Sigma‐Aldrich (MI, USA). Cycloheximide (CHX) was purchased from Aladdin (Shanghai, China). HIF‐1a overexpression plasmid and the overexpression control plasmid were produced and purchased from Genechem (Shanghai, China). Preparation of the osteogenic induction medium: To DMEM complete media, we added 10 mmol/L β‐Glycerol phosphate disodium salt (Solarbio, Beijing, China), 0.1 μmol/L dexamethasone (Solarbio, Beijing, China) and 50 mg/L ascorbic acid (Solarbio, Beijing, China), used after sterilizing with a 0.22 μm colander (Becton Dickinson, Franklin Lakes, NJ). Prepare the low‐oxygen inducing medium with a concentration of 200 μM CoCl2: 0.0065 g CoCl_2_ was added into 250 mL DMEM complete medium, shaken and mixed, and used in a 0.22 μm filter (Becton Dickinson, Franklin Lakes, NJ, USA) after degerming.^35^

### 2.2 Animals and ethical approval

All surgical interventions and postoperative animal care were performed according to the National Research Council's Guide for the Care and Use of Laboratory Animals and were approved by the Southeast University's Animal Research Ethics Committee (No. 20220620010). The experimental design aimed to minimize the number of animals used and sacrificed. Male Sprague–Dawley (SD) rats (6 weeks old, 250–300 g) were purchased from the Shanghai Family Planning Research Institute Laboratory Animal Management Department (Shanghai, China). The rats were housed in a humidity‐controlled room at 25°C, under a 12 h light/dark cycle, with free access to food and water.

### 2.3 Establishment of a diabetic rat model

The rats were fed a high‐sugar and fat diet for 2 months. The rats were weighed after overnight fasting. Further, 1% streptozotocin dosed at 40 mg/kg was prepared with a citric acid buffer solution based on fasting weight and quickly administered into the abdomen, which was completed within 30 min. After 3 days, blood glucose concentration in the rat tail vein was measured using a glucose meter. More details are outlined in a previous study.^36^ Figure 7A shows the flowchart of the animal experiments.

### 2.4 Establishment of a rat model of diabetic calcific tendinopathy

After successfully establishing the diabetic rat model,^36^ the rats were divided into four groups. They were Non‐DM+ normal saline (NS) (healthy rats injected with NS in bilateral Achilles tendon), DM + NS (diabetic rats injected with NS in bilateral Achilles tendon), DM + H_2_O_2_ (diabetic rats injected with 800 μmol/L H_2_O_2_ in bilateral Achilles tendon) and DM + H_2_O_2_ + NAC groups (diabetic rats injected with a mixture of 800 μmol/L H_2_O_2_ and 10 mM NAC into the bilateral Achilles tendon). Each group comprised eight rats. All rats received a single injection of 0.2 mL solution into the unilateral Achilles tendon twice a week for 8 weeks. Figure 7A shows a flowchart of the animal experiment.

### 2.5 Extraction and culture of TSPCs isolated from healthy rats

In this study, a mature and perfect extraction method developed by our research group was used for TSPCs extraction.^11^ The steps were as follows: Four 6‐week‐old healthy SD rats were selected, weighing 270–330 g. After the rats were euthanized, the middle part of the Achilles tendon was dissected from both sides, and the connective tissue around the Achilles tendon was carefully removed. Type I collagenase (3 mg/mL; Sigma‐Aldrich, St. Louis, MO, USA) digested collagen for 1.5 h. A 70 μm filter (Becton Dickinson, Franklin Lakes, NJ) formed a single‐cell suspension, and the cells were inoculated at a low density (50 cells/cm^2^) and cultured for 7–10 days. After clonal formation, pancreatic enzyme digestion was mixed and labelled as primary cells (P0). Meanwhile, we verified the self‐renewal capacity and multipotent differentiation potential of the extracted primary cells (Figure S1). P3–5 cells were used in follow‐up experiments. The culture medium was changed every 3 days during the experiment.

### 2.6 In vitro cell culture condition

All TSPCs cells were extracted from healthy SD rat Achilles tendons. Based on the development and progression of calcific tendinopathy, the TSPCs environment is divided into three stages: (1) Low oxygen and low glucose (LO–LG) levels represent the environment of tendon cells in healthy individuals without diabetes. We simulated TSPCs using a low‐glycemic complete medium containing 200 μM CoCl_2_.^35^ (2) Low oxygen and high glucose (LO–HG): In the early stages of diabetes, the blood glucose level increases; however, the tendon does not show vascularization, thickening, erythrocyte increase, or tissue oxygen concentration change. To simulate the intervention, a high‐glucose complete medium containing 200 μM CoCl_2_ was used. (3) High oxygen and high glucose (HO–HG) represent the progressive stage of diabetes when tendons have increased blood vessels and thickening, red blood cells, and tissue oxygen concentration. The cells were placed in a high‐glucose complete medium in an incubator with a 20% oxygen concentration for the intervention simulation.

### 2.7 Quantitative real‐time polymerase chain reaction (qRT‐PCR) assay

Total ribonucleic acid (RNA) was extracted from TSPCs using TRIzol reagent (Invitrogen, Carlsbad, CA, USA). RNA concentration and purity were measured using a spectrophotometer (NanoQTM; Beijing, China). The template RNA had an OD260/OD280 ratio ranging from 1.9 and 2.0. According to the manufacturer's user guide, complementary genes were synthesized by reverse transcription of 2 μg template RNA in a two‐step RT‐PCR SuperMix Kit (TransGen Biotech, China).^37^ Subsequently, a reverse transcription polymerase chain reaction was performed using the ABI7500 system (Thermo Fisher Scientific, USA). Finally, the messenger RNA (mRNA) expression of the gene was detected by 2‐ΔΔCt. See Table S1 for the primer sequences used.

### 2.8 Western blot (WB) assay

We evaluated the expression of VEGF, Hypoxia inducible factor‐1a(HIF‐1α), Alkaline phosphatase (ALP), Osteopontin (OPN), Osteocalcin (OCN), Osteonectin (ON), Runt‐related transcription factor 2 (RUNX2), Ubiquitin, and other proteins in the whole‐cell lysate products. Cells from each group were collected, and radio‐immunoprecipitation assay protein lysate (Invitrogen, Carlsbad, CA, USA) was added to the ice for full cracking. The cells were centrifuged at 4°C and 12,000 rpm, and the supernatant was collected. Using a BCA Protein Analysis Kit (Beyotime Biotechnology, Shanghai, China), the protein concentration in the supernatant was thoroughly mixed with sodium dodecyl sulfate‐polyacrylamide gel electrophoresis (SDS‐PAGE) protein loading buffer (5×) (Beyotime Biotechnology, Shanghai, China) at a 4:1 volume ratio. The resulting protein was heated at 100°C for 10 min for denaturation. Using 4%–20% SDS‐PAGE (ACE Biotechnology, Beijing, China), the same amount of protein (30 mg system) was transferred to the polyvinylidene fluoride (PVDF) membrane (Sigma, USA). The remaining experimental steps referred to previous experiments.^38^ ImageJ software was used to analyse the relative band strengths. The antibody information is listed in Table S2.

### 2.9 Co‐immunoprecipitation (Co‐IP) assay

TSPCs were cultured for 3 days in a 10 cm‐diameter cell culture dish in a high‐oxygen environment. The cell culture medium was removed, washed once with PBS, and cells were lysed with 500 μL of cell lysate (Invitrogen, Carlsbad, CA, USA). Approximately 1 μg of protein samples was obtained and mixed with 200 μL of immunoglobulin G (IgG) precipitation solution. The mixture was incubated with Protein A + G Agarose (Beyotime Biotechnology, Shanghai, China), containing 20 species of the same general IgG. The incubation was conducted at 4°C with gentle shaking for 1 h. The supernatant was collected for immunoprecipitation after centrifugation at 2500 rpm for 5 min. Two micrograms of ubiquitin or HIF‐1a primary antibody were added and slowly shaken overnight at 4°C. The next day, 40 μL of fully resuspended Protein A + G Agarose was added and slowly shaken at 4°C for 3 h. Following instantaneous high‐speed centrifugation, the supernatant was carefully removed, washed, and precipitated with 500 μL PBS five times. After the final washing, the supernatant was removed and added to the Vortex of 20–40 μL 1X SDS‐PAGE loading buffer for re‐suspension precipitation, and the sample was centrifuged to the bottom of the tube at instantaneous high speed. After treatment at 100°C for 5 min, some or all samples were subjected to SDS‐PAGE.

### 2.10 Transfection of TSPCs assay

A day before transfection (18–24 h), approximately 200,000–700,000 cells per well were inoculated into a six‐well plate for a culture so that the cell density reached approximately 70–80% on the following day. Before transfection, 2 mL of fresh culture solution was added to each well of a six‐well plate containing the cells. A clean, sterile centrifuge tube was obtained. Next, 125 μL of DMEM low‐ or high‐glucose media without antibiotics and serum was added to cells from each well of the six‐well plate to be transfected. Furthermore, 2.5 μg of HIF‐1a carrying GV657 vector (GV657‐HIF‐1A) plasmid (GeneChem, Shanghai, China) was transfected with the empty vector GV657 as the negative control, gently blown and mixed with a gun. Next, a transfection reagent was added to the 4 μL Lipo8000™ (Beyotime Biotechnology, Shanghai, China). After preparation, the samples were stored at room temperature for 6 h for stabilization. At 125 μL of the Lipo8000™ transfection agent‐DNA mixture per well of a six‐well plate, drops were evenly added to the entire well and gently mixed. Approximately 48 h after continuous culture, fluorescence microscopy was used to detect the transfection effect and the transfected cells were successfully used for follow‐up.

### 2.11 ALP staining and quantification assay

After 1 week of cell culture in each group, the culture medium was removed and the cells were washed once with PBS. An ALP staining kit (Solarbio, Shanghai, China). According to the manufacturer's instructions, the ALP‐fixing solution was added for 3 min and distilled water was used for cleaning. The prepared ALP solution was placed in a wet box, incubated for 20 min in the dark and cleaned with distilled water. A nuclear solid red staining solution was added and re‐dyed for 3 min. Cleaning with PBS, observation and detection was performed under a light microscope. An ALP detection kit (Nanjing Jiancheng Bioengineering Institute) was used for quantitative detection. Relevant reagents were added according to the manufacturer's instructions, and an enzyme marker measured the absorbance value of 520 nm of each well. Finally, the ALP activity was calculated using a correlation formula.

### 2.12 Alizarin red staining (ARS) and quantification assay

After 3 weeks of cell culture in each group, the culture medium was removed, and the cells were washed once with PBS. ARS staining kits (Beyotime Biotechnology, Shanghai, China) were used according to the manufacturer's instructions to join the fixed liquid for 20 min, followed by washing three times with PBS. An appropriate amount of ARS solution was added, and the cells were evenly covered and stained at room temperature for 30 min. The cells were washed thoroughly with distilled water, viewed and photographed under a microscope. To quantify ARS in 96‐well plates, we added 100 mmol/L cetylpyridine chloride (C9002; Sigma) solution (100 μ/L) and cetylpyridine chloride solution as blank. Optical density was measured at 405 nm using a Hitachi U‐2800A spectrophotometer.^39^

### 2.13 Transmission electron microscope inspection assay

TSPCs were cultured continuously for 2 weeks in LO–LG, LO–HG and HO–HG environments. The cells were washed once with PBS, hung with a cell spatula, centrifuged at 1500–3000 RPM for 5–10 min, and two to three rice‐sized samples were collected from the cells at the bottom of the tubes. The supernatant was discarded. Electron microscopic fixation solution (Servicebio, Wuhan, China), precooled at 4°C was slowly added along the tube wall, and the structure of organelles was observed by transmission electron microscope (Tecnai Spirit G2, FEI, USA).

### 2.14 ROS assay

Three days following each cell culture, the nutrient solution was removed, and the cells were washed with PBS using the active oxygen detection kit (Beyotime Biotechnology, Shanghai, China) to detect the contents of intracellular ROS, in accordance with the manufacturer's instructions to join the appropriate dilution volume of good DCFH‐DA, which was added to the positive control well. The cells were then incubated at 37°C for 20 min. The cells were washed thrice with a serum‐free cell culture solution to remove dichloro‐dihydro‐fluorescein diacetate that did not enter the cells completely. Finally, inverted fluorescence microscopy (Leica DM IL LED Fluo, Leica Microsystems, Germany) or flow cytometry (USA, Beckmancoulter, USA) was used to detect the fluorescence intensity in each group.

### 2.15 Lipid peroxidation malondialdehyde (MDA) assay and total antioxidant capacity assay

The MDA (Beyotime Biotechnology, Shanghai, China) and total antioxidant capacity (T‐AOC) assay kits (Beyotime Biotechnology, Shanghai, China) were used according to the manufacturer's instructions. The detection reagents were gradually added, and the lipid oxidation capacity and T‐AOC of each group of cells were calculated according to relevant formulas.

### 2.16 Immunocytofluorescence (ICF) assay

The cell medium in each group was removed, and the cells were washed with PBS thrice. Cold polyformaldehyde (4%; Biosharp, Hefei, China) was fixed for 15 min, washed three times with PBS for 5 min each time and the bed was shaken. Triton X‐100 (0.25%) (Sigma‐Aldrich, USA) was used to break the film for 15 min, washed thrice in PBS for 5 min each time, and the bed was shaken. The immune dyeing sealing fluid (Beyotime Biotechnology) was incubated for 60 min. Primary antibody (primary antibody diluent mixture) was added for incubation, and the bed was shaken overnight at 4°C. The primary antibody was collected, washed thrice with PBS for 5 min each time and the bed was shaken. Secondary antibodies, Alexa Fluor 488 and 594 (Proteintech, Wuhan, China), were incubated at room temperature for 60 min (in the dark). The secondary antibody was recovered, washed three times with PBS, and the bed was shaken for 5 min each time. 4′,6‐diamidino‐2‐phenylindole (DAPI) (Sigma‐Aldrich, USA) was incubated for 10 min (in the dark). The bed was washed three times with PBS for 5 min each. Detection was performed using an inverted fluorescence microscope (Leica DM IL LED Fluo; Leica Microsystems, Germany).

### 2.17 Micro‐computed tomography (Micro‐CT) assay

After anaesthesia, bilateral Achilles tendons were used for micro‐CT detection (SkyScan 1176, Bruker, Karlsruhe, Germany)^39^ to evaluate the calcification of bilateral Achilles tendons in rats. The X‐ray tube settings were 60 kV and 134 μA, and images were acquired at 50 μm resolution. A 0.5° rotation step through a 360° angular range with 50 ms exposure per step was used. The images were reconstructed using the Hiscan reconstruct software (Bruker, Karlsruhe, Germany). The Hiscan Analyser software (Bruker, Karlsruhe, Germany) was used to analyse the ossified area parameters, including bone volume and CT values.^39^, ^40^

### 2.18 Haematoxylin and eosin staining assay

The rats were euthanized, and the tendon samples were immediately removed and fixed in 10% neutral formalin (Servicebio, China) for 24 h. The fixed samples were then gradually dehydrated in increasing concentrations of ethanol, typically 70%, 80%, 90% and 100% ethanol, to remove water from the tissue. The dehydrated samples were subsequently immersed in xylene (Aladdin, China) to further remove alcohol and make the tissue samples compatible with paraffin. The samples were then placed in molten paraffin to ensure complete infiltration. After paraffin infiltration, the samples were embedded in moulds with molten paraffin to form paraffin blocks. The paraffin blocks were sectioned into 3‐micron thick tissue slices using a microtome (CUT4050, Micro Tec, Germany). The slices were then mounted on slides and subjected to subsequent staining. Paraffin sections were dewaxed in water, stained with haematoxylin (Nanjing Yorm Biotechnology, China) for 5 min, and washed with running water to remove excess dye. The sections were stained with eosin (Nanjing Yorm Biotechnology, China) for 1 min and washed with tap water to remove excess dye. The slices were then immersed in anhydrous ethanol for 2 min and xylene for 5 min. Each section was removed, an appropriate amount of neutral gum was added to the section, and the slide was covered with a scanner (VS200, OLYMPUS, Japan) and scanned at 20× objective.

### 2.19 Masson staining assay

The paraffin embedding and sectioning of tendon samples were performed according to the H&E staining protocol. The paraffin sections were dewaxed in water, immersed in Bouin's solution (Kohypath, Shanghai, China), and fixed overnight at room temperature. The slices were immersed into Masson staining Kit A solution (Nanjing Yorm Biotechnology, China), stained at room temperature for 10 min, washed with tap water, and stained blue. Sections were immersed in Masson staining Kit B solution, stained at room temperature for 8 min and washed with tap water. The sections were divided into Masson staining kit C solution, differentiated at room temperature for 5 min, and excess liquid was drained. The sections were immersed in Masson's staining kit D solution, stained at room temperature for 3 min, and washed with tap water. Next, the slices were added to 0.2% glacial acetic acid and washed thoroughly. The slices were then immersed in anhydrous ethanol for 2 min and xylene for 5 min. Each section was removed, an appropriate amount of neutral gum was added and the slide was covered with a scanner (VS200, OLYMPUS, Japan) and scanned at 20× objective.

### 2.20 Immunohistofluorescence (IHF) assay

The paraffin embedding and sectioning of tendon samples were performed according to the H&E staining protocol. The paraffin sections were dewaxed in water, and the high‐temperature‐resistant plastic dye stand containing the sections was placed in a repair box filled with citric acid antigen repair solution (pH 6.0) and heated at medium‐high temperatures for 30 min. The solution was cooled naturally to room temperature and soaked in PBS thrice for 5 min each. In each group, 3% of blood serum albumins (BSA) (Beyotime Biotechnology, Shanghai, China) in closed drops was added for uniform coverage, followed by a 30 min incubation at room temperature. After the BSA (Beyotime, China) sealer was removed, an appropriate amount of the primary antibody working liquid diluted with PBS was added to the tissue, and the sections were placed flat in a wet box and incubated overnight at 4°C. The slices were removed the following day and reheated at room temperature for 30 min. Soak in PBS three times for 5 min each time. An appropriate amount of the secondary antibody was added to the tissue and incubated at room temperature for 1 h. Subsequently, each section was soaked in PBS three times for 5 min each time. The sections were immersed in an autofluorescence quencher A solution and incubated at room temperature for 30 min. Next, each section was soaked in PBS three times for 5 min each time. An appropriate amount of DAPI solution (Sigma‐Aldrich, USA) was added to the tissues and incubated at room temperature for 10 min in the dark. Each section was soaked in PBS three times for 5 min each time. An appropriate amount of anti‐quenching sealing glue was added to the tissue to cover the glass. Each section was removed, an appropriate amount of neutral gum was added and the slide was covered with a scanner (VS200, OLYMPUS, Japan) and scanned at 20× objective.

### 2.21 Immunohistochemistry (IHC) assay

The paraffin embedding and sectioning of tendon samples were performed according to the H&E staining protocol. The paraffin sections were dewaxed in water, and the high‐temperature‐resistant plastic dye stand containing the sections was placed in a repair box filled with citric acid antigen repair solution (PH6.0) and heated at medium‐high temperatures for 30 min. The solution was cooled naturally to room temperature and soaked in PBS thrice for 5 min each. A hydrophobic pen was used to draw a circle around the tissue. An appropriate amount of 3% BSA blocking solution (Beyotime, China) was applied to each tissue block, ensuring that the tissue was evenly covered and incubated at room temperature for 30 min. The BSA blocking solution (Beyotime, China) was then flicked off, and an appropriate amount of PBS‐diluted primary antibody working solution RUNX‐2 (Proteintech, China) and OCN (Proteintech, China) were applied to the tissue, covering it evenly. The slides were placed flat in a humidified chamber and incubated at 4 C overnight. All other experimental procedures have been described in previous studies.^41^ Five fields of the same size were randomly selected from each group and the mean optical density (MOD) of immunohistochemical positive parts was analysed by Image J 1.51j8 (NIH, USA).

### 2.22 Statistical analysis

Data are presented as the mean ± standard deviation (SD). IBM SPSS statistics software (version 26.0, SPSS Inc., Chicago, United States) and GraphPad Prism software (version 8.4.0, GraphPad Software, Boston, United States) were used to analyse the data. After analysing the normality using the Shapiro–Wilk test, data between two groups were evaluated by the *t*‐test and nonparametric test. For three and more groups, data were evaluated by one‐way ANOVA. *p < 0.05, **p<0.01, ***p<0.001 and ****p<0.0001 indicated significant differences.

## 3 RESULTS

### 3.1 Hyperoxia promoted osteogenic differentiation and inhibited tenogenic differentiation of TSPCs

**FIGURE 2:**
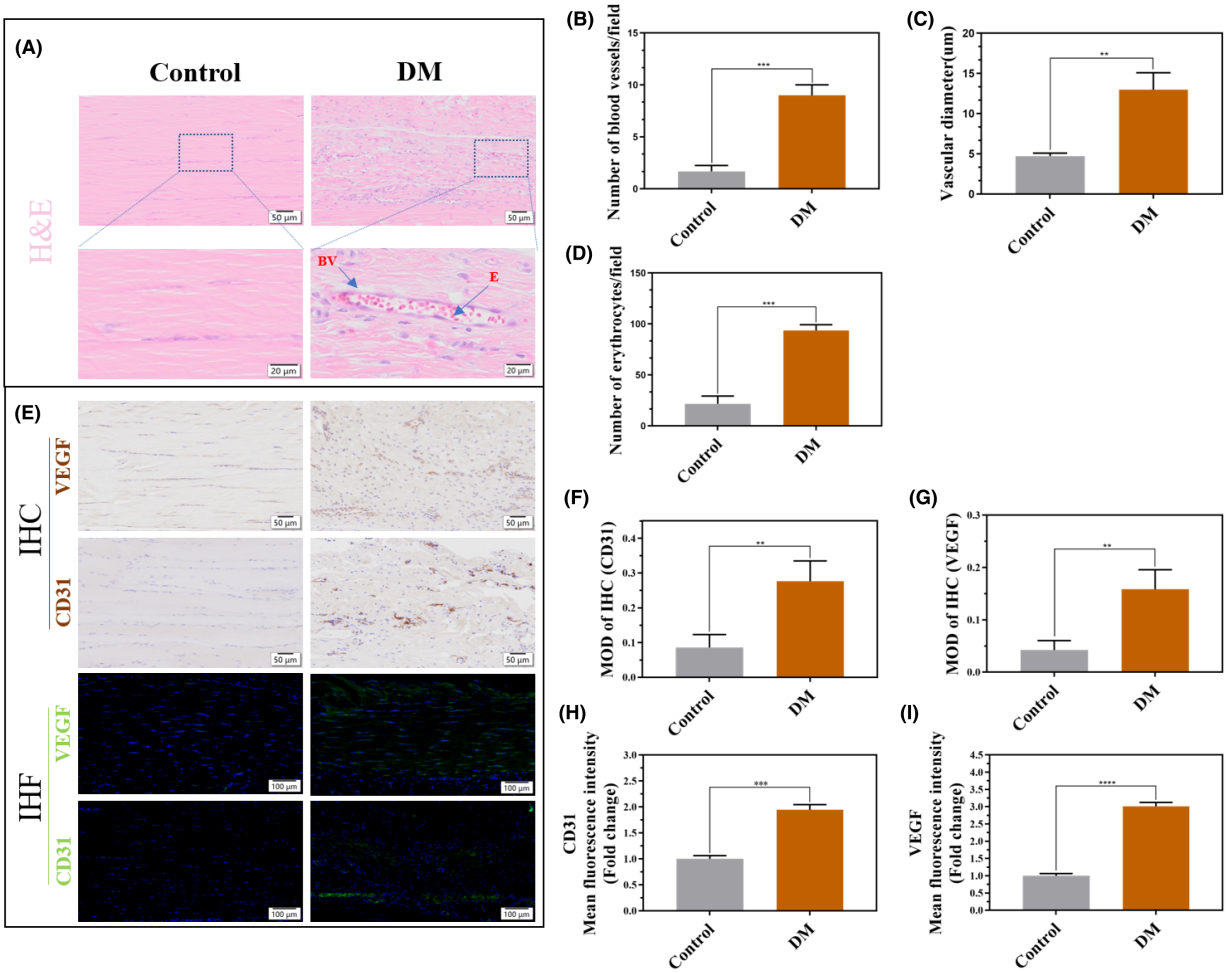
The quantity and structure of blood vessels and haemoglobin in the Achilles tendon tissue of diabetic rats changed. (A) Diabetic rats were fed for 4 weeks after successful modelling. H&E staining was used to observe changes in the morphology and quantity of blood vessels and haemoglobin in the Achilles tendon of the two groups. (B–D) The number and diameter of blood vessels and the quantity of haemoglobin were quantitatively analysed. (E) IHC and IHF were used to detect the expression of the vascular active factors VEGF and CD31 in the Achilles tendon tissues in each group. (F‐I) Semi‐quantitative analyses of VEGF and CD31 proteins were performed. Data are presented as the mean ± standard deviation. *n* = 3 for each group, **p<0.01, ***p<0.001, ****p<0.0001. Scale bars: 20, 50, and 100 μm. DM, Diabetes mellitus, H&E, Haematoxylin and eosin, IHC, Immunohistochemistry, IHF, Immunohistofluorescence, VEGF, Vascular endothelial growth factor.

Histological analysis showed increased blood vessels, red blood cells, and tendon thickening in the Achilles tendon tissues of diabetic rats (Figure 2A), and the differences were statistically significant by semi‐quantitative analysis (Figure 2B–D). The expression of the vascular‐related factors, VEGF and CD31, in the Achilles tendon tissues of diabetic rats was significantly increased, as observed after IHC and IHF staining (Figure 2E–I). The qRT‐PCR results showed that VEGF mRNA expression in TSPCs was significantly increased in the LO‐HG group (*p*<0.001; Figure S2A). WB (*p*<0.0001; Figure S2B,C) and ICF (*p*<0.01; Figure S2D,E) results also showed that the VEGF protein levels were significantly increased in the LO‐HG group.

**FIGURE 3:**
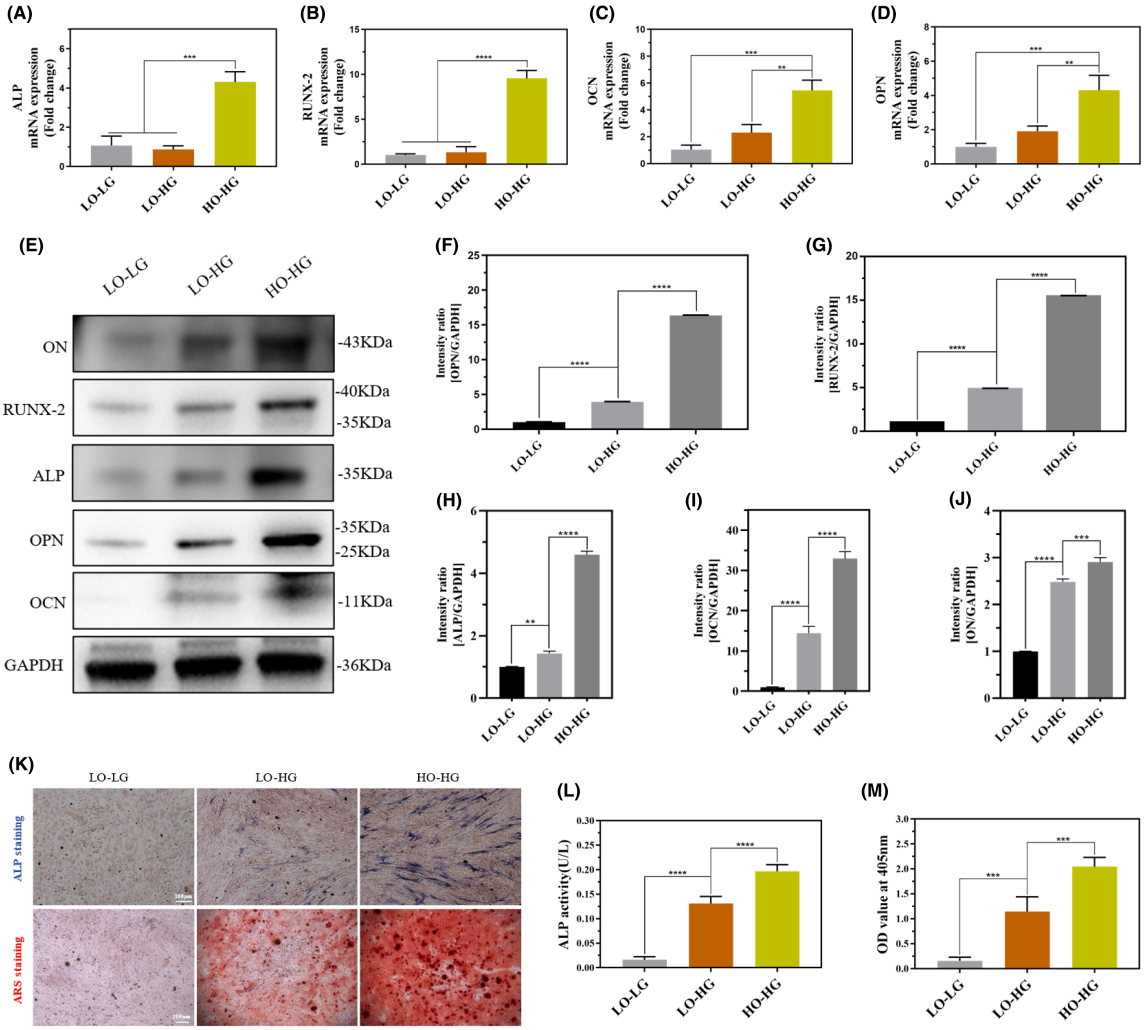
Hyperoxia promoted osteogenic differentiation of TSPCs. (A–D) TSPCs isolated from healthy rats were cultured with LO‐LG, LO‐HG, and HO‐HG for 2 weeks. The mRNA expression levels of ALP, RUNX‐2, OCN, and OPN were detected using qRT‐PCR. (E–J) TSPCs were cultured for 3 weeks. The protein expression levels of RUNX‐2, OPN, OCN, ALP and ON were detected by WB. Consequently, a semi‐quantitative analysis of the WB results was performed. All full‐length blots are presented in File S2: Figure 3E. (K–M) TSPCs were cultured for 1 week. ALP staining and quantitative analysis were performed. TSPCs were cultured for 3 weeks, and ARS staining and quantitative analysis were performed. Data are presented as the mean ± standard deviation. *n* = 3 for each group, **p<0.01, ***p<0.001, ****p<0.0001. Scale bars: 100 and 200 μm. LO‐LG, Low oxygen and low glucose, LO‐HG, Low oxygen and high glucose, HO‐HG, High oxygen and high glucose, TSPCs, Tendon stem/progenitor cells, OPN, Osteopontin, OCN, Osteocalcin, ON, Osteonectin, WB, Western blot, ALP, Alkaline phosphatase, ARS, Alizarin red staining.

TSPCs were cultured under the following three conditions: LO‐LG, LO‐HG and HO‐HG. The qRT‐PCR results showed that the mRNA expression of the osteogenic genes ALP, RUNX‐2, OCN and OPN in TSPCs in the HO‐HG group was higher than that in the LO‐LG and LO‐HG groups (Figure 3A–D). WB (*p*<0.0001; Figure 3E–J) and ICF (Figure S3) also showed that the protein expression levels of RUNX‐2 and OCN in TSPCs were significantly increased in the HO–HG group. The positive rates of osteogenic ALP and ARS staining in TSPCs were enhanced in the HO–HG group (Figure 3H–J). However, the mRNA expression of the tenogenic genes mohawk (MKX), scleraxis (SCX), tenascina (TNC), tenomodulin (Tnmd), and collagen type III (COL‐3) in TSPCs significantly decreased in the HO–HG group (Figure S4A–E). The protein expression levels of SCX, COL‐3 in TSPCs significantly decreased in the HO–HG group (Figure S4F–H).

### 3.2 Hyperoxia regulated ROS/HIF‐1a signalling axis activity and mediated HIF‐1a protein ubiquitination degradation

**FIGURE 4:**
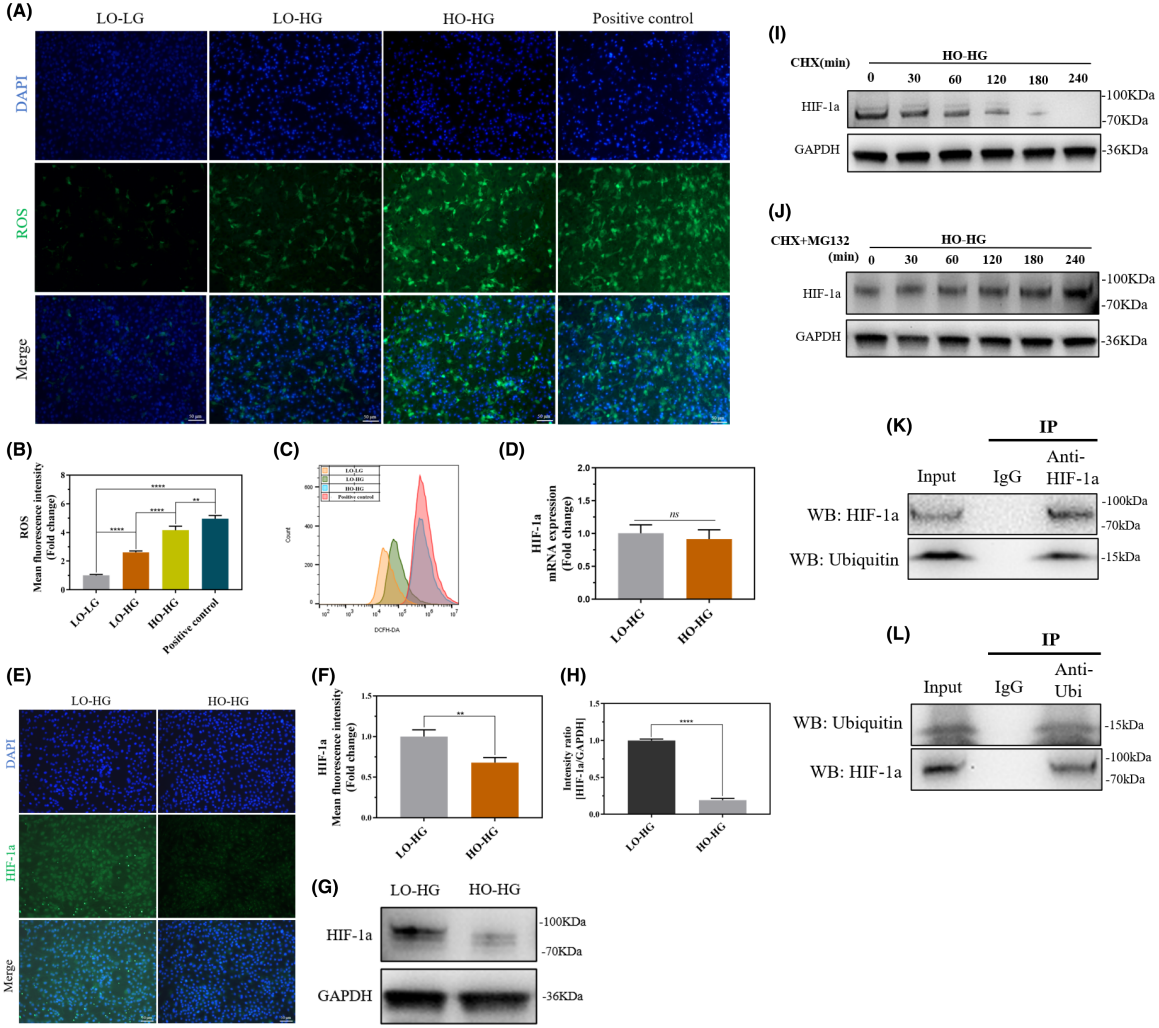
Changes in the ROS/HIF‐1a signalling axis caused by hyperoxia. (A, B) TSPCs were cultured for 3 days. ROS content in each group was detected by fluorescence microscopy, and a semi‐quantitative analysis was performed. (C) ROS content in each group was detected by flow cytometry. (D) The mRNA expression of HIF‐1a in each group was detected by qRT‐PCR. (E, F) TSPCs were cultured for 3 days, and the protein expression level of HIF‐1a in each group was detected by IF. Consequently, a semi‐quantitative analysis was performed. (G, H) The protein expression level of HIF‐1a in each group was detected by WB. All full‐length blots are presented in File S2: Figure 4G. (I, J) CHX and MG132 were added to the HO‐HG environment of TSPCs, and the protein expression level of HIF‐1a in TSPCs was detected by WB at 0, 30, 60, 120, 180, and 240 min. All full‐length blots are presented in File S2: Figure 4I,J. (K, L) The combination of HIF‐1a and ubiquitin in TSPCs was detected by Co‐IP after culturing in HO‐HG for 3 days. All full‐length blots are presented in File S2: Figure 4K,L. Data are presented as the mean ± standard deviation. *n* = 3 for each group, ns: Not significant, **p<0.01, ****p<0.0001. Scale bar: 50 μm. LO‐LG, Low oxygen and low glucose, LO‐HG, Low oxygen and high glucose, HO‐HG, High oxygen and high glucose, TSPCs, Tendon stem/progenitor cells, ROS, Reactive oxygen species, WB, Western blot, qRT‐PCR, Quantitative real‐time polymerase chain reaction.

TSPCs induced intracellular ROS changes in the LO–LG, LO–HG, and HO–HG groups. Fluorescence results showed that ROS expression was significantly increased in the HO–HG group (Figure 4A,B), and the flow cytometry results showed that the peak value shifted significantly to the right in the HO–HG group (Figure 4C). TEM results showed that the mitochondria were short and rod‐like in the LO‐LG group (Figure S5A), and swelled and round in the LO‐HG group (Figure S5B). Morphological and structural changes in the mitochondria were more obvious in the HO‐HG group (Figure S5C). In addition, MDA (lipid oxidation) capacity was significantly increased in the HO‐HG group (Figure S5D), while T‐AOC significantly decreased (Figure S5E).

qRT‐PCR showed no significant differences in HIF‐1a gene expression (*p* = 0.4591; Figure 4D). But, ICF (*p*<0.01; Figure 4E,F) and WB (*p*<0.0001; Figure 4G,H) results showed that HIF‐1a protein expression was significantly lower in the HO‐HG group than in the LO‐HG group. To verify whether the proteasome degraded HIF‐1a, 100 μg/mL CHX was added to TSPCs cultured in an HO‐HG environment to block ribosomal protein synthesis. Over time, the expression of HIF‐1a protein gradually decreased (Figure 4I). On this basis, 40 mM MG132 was added simultaneously. The protein expression of HIF‐1a did not show a significant reduction over time (Figure 4J). To further verify whether HIF‐1a degradation is caused by protein ubiquitination, CO‐IP experiments were conducted on TSPCs cultured in an HO‐HG environment, and the results showed that an interaction between HIF‐1a and Ubiquitin (Figure 4K,L).

### 3.3 ROS/HIF1‐a signalling axis mediated osteogenic differentiation of TSPCs

**FIGURE 5:**
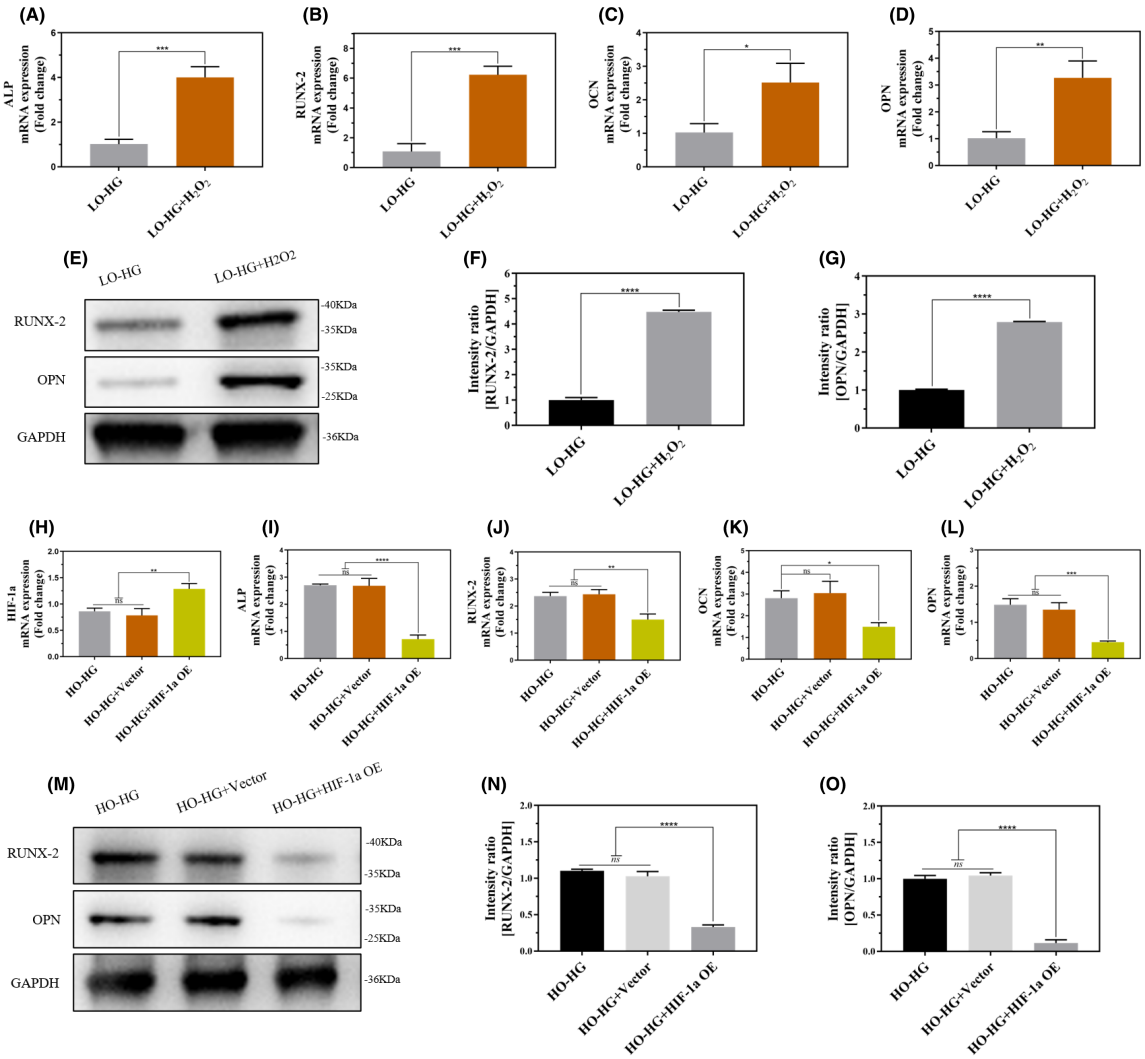
ROS/HIF‐1a signalling axis mediated osteogenic differentiation of TSPCs. (A–D) TSPCs were cultured with LO‐HG and LO‐HG + H_2_O_2_ for 2 weeks. The mRNA expression levels of the osteogenic genes ALP, RUNX‐2, OCN, and OPN in TSPCs were detected by qRT‐PCR. (E–G) The protein expression levels of RUNX‐2 and OPN in TSPCs were detected by WB. Consequently, a semi‐quantitative analysis of WB results was performed. All full‐length blots are presented in File S2; Figure 5E. (H) Transfection efficiency of HIF‐1a overexpressed plasmid in TSPCs was detected by qRT‐PCR after transfection for 48 h. (I–L) After transfection, the mRNA expression levels of the osteogenic genes ALP, RUNX‐2, OCN, and OPN were detected by qRT‐PCR. (M–O) The protein expression levels of RUNX‐2 and OPN were detected by WB. Semi‐quantitative analysis of WB was performed. All full‐length blots are presented in File S2; Figure 5M. ns, Not significant, *p<0.05, **p<0.01, ***p<0.001, ****p<0.0001. Data are presented as the mean ± standard deviation. *n* = 3 for each group. ns, Not significant, *p<0.05, **p<0.01, ***p<0.001, ****p<0.0001. LO‐HG, Low oxygen and high glucose, HO‐HG, High oxygen and high glucose, H_2_O_2_, Hydrogen peroxide, TSPCs, Tendon stem/progenitor Cells, mRNA, Messenger RNA, ALP, Alkaline phosphatase, OCN, Osteocalcin, OPN, Osteopontin, qRT‐PCR, Quantitative real‐time polymerase chain reaction, WB, Western blot, HIF‐1a, Hypoxia‐inducible factor‐1a.

In this study, ROS promoted the osteogenic differentiation of TSPCs. ROS levels were lower in the LO‐HG group than in the HO‐HG group (*p*<0.0001; Figure 4A–C). In the presence of 400 μmol/L H_2_O_2_ in the LO‐HG medium, the expression of TSPCs osteogenic genes ALP, RUNX‐2, OCN, OPN (Figure 5A–D) and proteins RUNX‐2, OPN (*p*<0.0001; Figure 5E–G) were significantly increased in the LO‐HG + H_2_O_2_ group compared to the LO‐HG group.

Additionally, HIF‐1a inhibits the osteogenic differentiation of TSPCs. Compared to that of the LO‐HG group, HIF‐1a protein expression was downregulated in the HO‐HG group (Figure 4E–H). After introducing the HIF‐1a overexpression plasmid into the HO‐HG culture environment, the transfection efficiency was assessed using qRT‐PCR (Figure 5H). The expression of TSPCs osteogenic genes ALP, RUNX‐2, OCN, OPN (Figure 5I–L), and proteins RUNX‐2, OPN (*p*<0.0001; Figure 5M–O) were significantly lower in the HO‐HG + HIF‐1a OE group.

Additionally, ROS regulates HIF‐1a protein expression. When 400 μmol/L H_2_O_2_ was added to the LO‐HG environment, no significant difference was observed in HIF‐1a gene expression between the LO‐HG and LO‐HG + H_2_O_2_ groups (*p* = 0.3399; Figure S6A). However, WB (*p*<0.0001; Figure S6B,C) and ICF (*p*<0.05; Figure S6D,E) results showed that the protein expression of HIF‐1a was significantly decreased in the LO‐HG + H_2_O_2_ group.

### 3.4 Hyperoxia promoted osteogenic differentiation of TSPCs through ROS/HIF‐1a pathway axis

**FIGURE 6:**
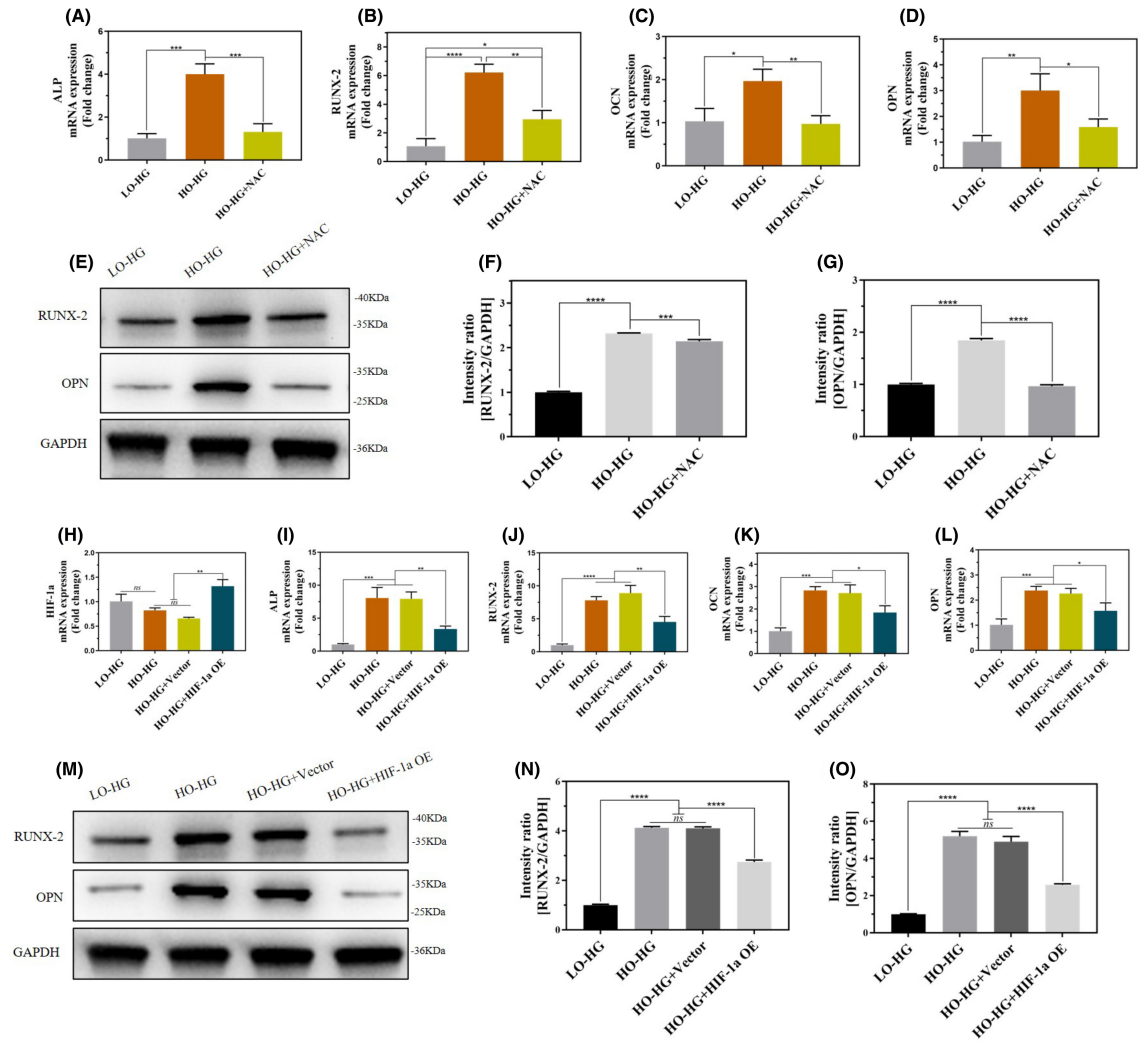
Hyperoxia promoted osteogenic differentiation of TSPCs through the ROS/HIF‐1a signalling axis. (A–D) NAC inhibited intracellular ROS content, and qRT‐PCR was used to detect the mRNA expression levels of the osteogenic genes ALP, RUNX‐2, OCN, and OPN in TSPCs. (E–G) The protein expression levels of RUNX‐2 and OPN in TSPCs were detected by WB. Consequently, a semi‐quantitative analysis of WB results was performed. All full‐length blots are presented in File S2: Figure 6E. (H) qRT‐PCR was used to determine the transfection efficiency of HIF‐1a overexpressed the plasmid in TSPCs after transfection with a plasmid for 48 h. (I–L) After transfection, TSPCs were cultured for 2 weeks. The mRNA expression levels of osteogenic genes ALP, RUNX‐2, OCN, and OPN in TSPCs were detected by qRT‐PCR. (M–O) After transfection, TSPCs were cultured for 3 weeks. The protein expression levels of osteogenic genes RUNX‐2 and OPN in TSPCs were detected by WB. Consequently, a semi‐quantitative analysis of WB was performed. All full‐length blots are presented in File S2: Figure 6M. Data are presented as the mean ± standard deviation. *n* = 3 for each group. ns: Not significant. *p<0.05, **p<0.01, ***p<0.001, ****p<0.0001. LO‐HG, Low oxygen and high glucose, HO‐HG, High oxygen and high glucose, NAC, N‐acetylcysteine, TSPCs, Tendon Stem/Progenitor Cells, ROS, Reactive Oxygen Species, qRT‐PCR, Quantitative real‐time polymerase chain reaction, OCN, Osteocalcin, OPN, Osteopontin, HIF‐1a, Hypoxia‐inducible factor‐1a, ALP, Alkaline phosphatase.

To verify whether hyperoxia promotes the osteogenic differentiation of TSPCs through the ROS/HIF‐1a pathway, we observed changes in the osteogenic differentiation of TSPCs by blocking ROS production and overexpressing the HIF‐1a gene. ROS expression was lower in the HO‐HG group than in the LO‐HG group (*p*<0.0001; Figure 4A–C). Further, the expression of osteogenic genes ALP, RUNX‐2, OCN, OPN (Figure 6A–D) and proteins RUNX‐2, OPN (*p*<0.0001; Figure 6E–G) in TSPCs were significantly increased in the HO‐HG group compared to the LO‐HG group. However, NAC was added to the HO‐HG environment to inhibit ROS production, and the results showed that the expression of the osteogenic genes ALP, RUNX‐2, OCN, OPN (Figure 6A–D), and proteins RUNX‐2, OPN (Figure 6E–G) in TSPCs were decreased in the HO‐HG + NAC group.

Additionally, protein expression of HIF‐1a of TSPCs was lower in the HO‐HG group compared to that in the LO‐HG group (Figure 4E–H). The expression of osteogenic genes ALP, RUNX‐2, OCN, OPN (Figure 6I–L), and proteins RUNX‐2, OPN (P<0.0001; Figure 6M–O) in TSPCs were significantly increased in the HO‐HG group compared to that in the LO‐HG group.

Nevertheless, following the introduction of the HIF‐1a overexpression plasmid into the HO‐HG environment, the transfection efficiency was detected by qRT‐PCR (Figure 6H), and the results showed that the expression of TSPCs osteogenic genes ALP, RUNX‐2, OCN, OPN (Figure 6I–L), and proteins RUNX‐2, OPN (*p*<0.0001; Figure 6M–O) were significantly decreased in the HO‐HG + HIF‐1a OE group.

### 3.5 Hyperoxia promoted calcification of Achilles tendon tissue in rats

**FIGURE 7:**
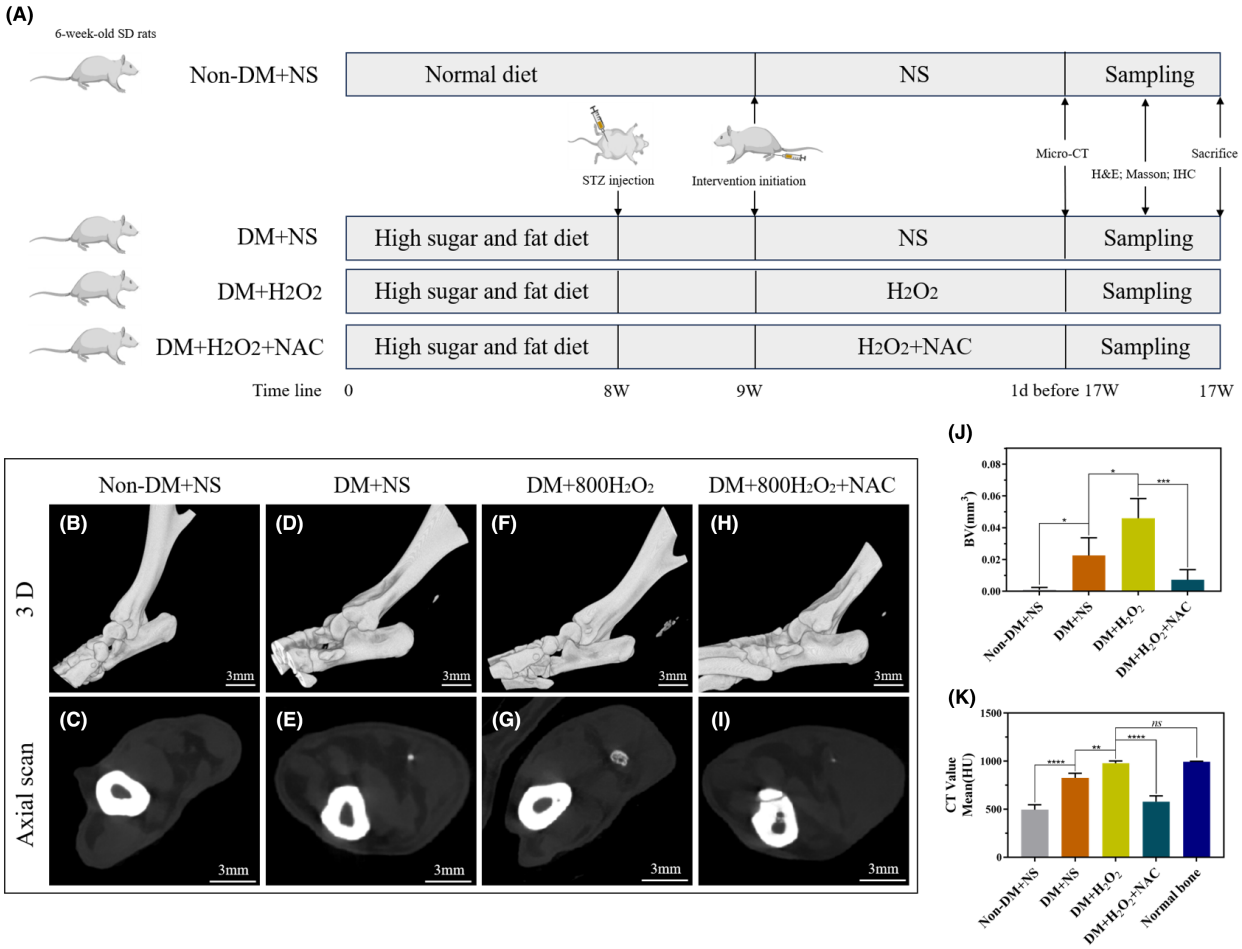
Hyperoxia promoted calcification in rat Achilles tendon tissue, whereas the antioxidant NAC inhibited the formation of calcification. (A) The flowchart of animal experimentation. (B–I) Micro‐CT was used to detect calcification in the rat Achilles tendon in the Non‐DM + NS, DM + NS, DM + H_2_O_2_, and DM + H_2_O_2_ + NAC groups after intervention for 8 weeks. (J–K) Quantitative analysis of bone volume (mm^3^) and CT Value (HU) in the four groups. Data are presented as the mean ± standard deviation. *n* = 3 for each group. ns, Not significant, *p<0.05, **p<0.01, ***p<0.001, ****p<0.0001. Scale bar: 3 mm. NAC, N‐acetylcysteine, Micro‐CT, Micro computed tomography, NS, Normal saline, H_2_O_2_, Hydrogen peroxide, DM, Diabetes mellitus, BV, Bone volume.

We conducted an 8‐week intervention in the Achilles tendon tissue of rats using various factors and examined the development of Achilles tendon calcification. Simultaneously, we added 10 mM NAC to evaluate its inhibitory effect on calcification in the Achilles tendon tissue (Figure 7A). After 8 weeks, micro‐CT results of the Achilles tendon tissue showed that compared with that in the non‐diabetic rats (Figure 7B,C), calcification appeared in the Achilles tendon of the DM group (Figure 7D,E) and the volume of calcified tissue in the Achilles tendon of diabetic rats and the calcification intensity significantly increased in the DM + H_2_O_2_ group (Figure 7F,G). After adding the antioxidant NAC, calcification in the Achilles tendon and the calcification intensity in the DM + H_2_O_2_ + NAC group decreased significantly (Figure 7H,I). Relevant quantitative analyses confirmed these trends (Figure 7J,K).

**FIGURE 8:**
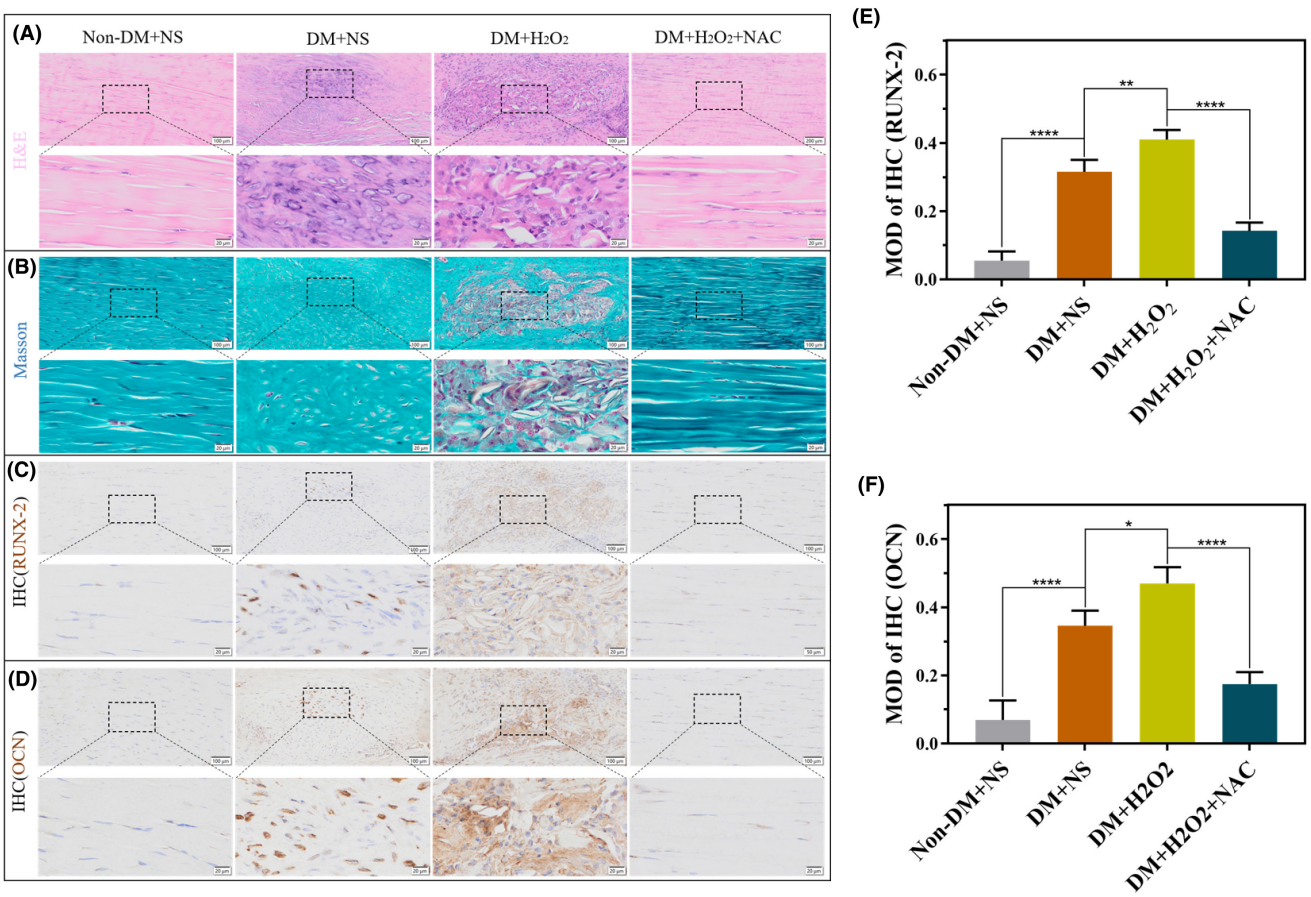
Calcification of the Achilles tendon was observed in the non‐DM + NS, DM + NS, DM + H_2_O_2_, and DM + H_2_O_2_ + NAC groups after intervention for 8 weeks. (A, B) H&E and Masson staining of the Achilles tendon at 8 weeks. (C, D) IHC of the Achilles tendon at 8 weeks. (E–F) Quantitative analysis of the protein expression of RUNX‐2 and OPN. Data are presented as the mean ± standard deviation. *n* = 3 for each group. ns: Not significant, *p<0.05, **p<0.01, ****p<0.0001. Scale bars: 20 and 100 μm. H&E, Haematoxylin and eosin, IHC, Immunohistochemistry, OCN, Osteopontin, H_2_O_2_, Hydrogen peroxide, DM, Diabetes mellitus, NAC, N‐acetylcysteine, NS, Normal saline.

H&E staining results showed that compared with those in non‐diabetic Achilles tendon tissues, the fibres in the Achilles tendon tissues of the DM + NS group were disordered and osteochondrocytes appeared, while the fibres in the Achilles tendon tissues of the DM + H_2_O_2_ group had more osteochondrocytes. Compared with that in the DM + H_2_O_2_ group, the arrangement of tendon fibres in the DM + H_2_O_2_ + NAC group were normal and osteochondrocyte numbers were significantly reduced (Figure 8A). Masson staining results showed that compared with those in the Achilles tendon tissues of non‐diabetic rats, the collagen fibres in the Achilles tendon tissues of the DM + NS group were relatively reduced and disordered, whereas the collagen fibres in the Achilles tendon tissues of the DM + H_2_O_2_ group were more significantly reduced and disordered. Compared to those in the DM + H_2_O_2_ group, the collagen fibres of the Achilles tendon were significantly increased and arranged more neatly in the DM + H_2_O_2_ + NAC group (Figure 8B). IHC results showed that the expression of RUNX‐2 and OCN were increased in the Achilles tendon tissues of the DM + NS group compared to that in non‐diabetic rats, whereas the expression of RUNX‐2 and OCN were more obvious in the Achilles tendon tissues of the DM + H_2_O_2_ group. Compared to those in the DM + H_2_O_2_ group, RUNX‐2 and OCN expressions in the Achilles tendon tissues of the DM + H_2_O_2_ + NAC group were significantly decreased (Figure 8C–F).

## 4 DISCUSSION

This study suggests that there may be a local hyperoxic environment in Achilles tendon tissue in diabetic rats compared to healthy rats. Under the stimulation of hyperoxia, the osteogenic differentiation ability of TSPCs was enhanced, and ROS/HIF‐1α signalling axis played a key role in the osteogenic differentiation process. Meanwhile, antioxidant NAC can effectively prevent osteogenic differentiation and Achilles tendon calcification of TSPCs.

Blood vessels and haemoglobin serve as conduits and carriers for oxygen transport within the body, and their abundance and morphological characteristics directly influence local oxygen concentrations in the organism. A greater density of blood vessels and erythrocytes signifies a higher local oxygen concentration.^42^, ^43^ In this study, compared to healthy rats, diabetic rats displayed an increased presence of blood vessels and haemoglobin within their Achilles tendon tissue, accompanied by the thickening of vascular cross‐sections. Moreover, the increased expression of VEGF and its processes in TSPCs suggests enhanced local oxygen concentration within the diabetic tendon tissue. Notably, Handa et al.^15^ identified significantly elevated levels of VEGF mRNA and protein expression in the shoulder cuff tissue of patients with diabetes compared to those of patients without. In addition, VEGF‐positive vessels exhibited greater numbers and larger cross‐sectional areas. Furthermore, our previous research indicated a pronounced increase in the number of blood vessels and intravascular red blood cells within the patellar tendon tissue of diabetic rats compared to that in healthy rats.^36^ These collective findings imply that prolonged exposure of tendons to a high‐glucose environment induces the upregulation of angiogenic substances, such as VEGF and CD31, triggering angiogenesis and an increase in haemoglobin content within the tendon tissue. This sequence of events leads to elevated local oxygen concentrations, ultimately culminating in the establishment of a high‐oxygen microenvironment within the tissues. In summary, based on both prior investigations and the present study, we pioneered the formation of a localized high‐oxygen environment within the diabetic tendon tissue.

Oxygen is a crucial component of cellular energy metabolism and plays a pivotal role in regulating numerous molecules and tissue functions. The results of this study revealed that, compared to a low‐oxygen environment, TSPCs demonstrated elevated expression of osteogenic differentiation‐related genes and proteins, along with enhanced cellular osteogenic processes under high‐oxygen conditions. Notably, Lee et al.^44^ found that hTSPCs exhibited a substantial increase in osteogenic differentiation potential in a 20% high‐oxygen environment. In our previous studies, we observed spontaneous osteogenic differentiation of rat TSPCs in a regular cell culture incubator (20% oxygen concentration),^45^ implying an augmented osteogenic potential of TSPCs in high‐oxygen environments. However, contrasting results were reported by Balogh et al.,^35^ who discovered that human aortic vascular smooth muscle cells (VSMCs) exhibited increased expression of osteogenic‐related genes and proteins, such as RUNX2, OCN, and ALP, accompanied by induction of the extracellular matrix, under low‐oxygen conditions (5% O_2_) compared to high‐oxygen conditions. Similarly, Mokas et al.^46^ found that VSMCs demonstrated enhanced expression of osteogenic differentiation‐related genes and proteins, as well as VSMC calcification under low‐oxygen conditions, contrary to our findings. We hypothesised that these discrepant outcomes stem from the distinct microenvironments in which TSPCs and VSMCs reside. TSPCs inhabit a low‐oxygen environment with minimal vasculature, while VSMCs exist in a high‐oxygen environment. Elevated oxygen levels alter the original low‐oxygen milieu of TSPCs, leading to a misdirection towards osteogenic differentiation.^47^ In contrast, VSMCs maintain their phenotype under high‐oxygen conditions, inhibiting osteogenic differentiation. This further indicates that in diabetic calcific tendon disease, the inherent low‐oxygen environment of TSPCs is disrupted and replaced by a high‐oxygen environment. Consequently, elevated oxygen levels enhance TSPC osteogenic differentiation, thereby promoting the occurrence and progression of diabetic calcific tendon diseases.

Previous research has highlighted the pivotal molecular regulatory role of the ROS/HIF‐1α signalling axis in governing stem cell osteogenic differentiation under high‐oxygen regulation. Here, this study indicated that high oxygen levels induce an increase in intracellular ROS and a reduction in HIF‐1α expression within TSPCs, and excess ROS also leads to a decrease in HIF‐1α expression. Zhang et al.^48^ also found that high oxygen levels can induce cellular damage through promoting the oxidative stress response in BMSCs. And a close correlation between cellular HIF‐1α expression and oxygen concentration was demonstrated, where high oxygen concentrations could suppress HIF‐1α expression.^49^, ^50^ Collectively, these previous findings are consistent with our study results. Additionally, we observed that the protein levels of HIF‐1α decreased without a significant alteration in gene expression under high‐oxygen conditions. Subsequently, ribosomal protein synthesis was inhibited, leading to a gradual reduction in protein degradation over time. Blocking proteasomes revealed no significant degradation of HIF‐1α protein, and molecular interactions between HIF‐1α and ubiquitin were observed. The ubiquitin‐proteasome system is a pivotal pathway for intracellular protein degradation that influences a range of cellular processes, and its dysfunction is associated with numerous human diseases.^51^, ^52^, ^53^ Kang et al.^54^ found that HIF‐1α in human cells under normoxic conditions was degraded via the ubiquitin‐proteasome system. Consistently, Tseng HI et al.^55^ revealed that the small molecule SCT‐1015 promotes HIF‐1α hydroxylation through AMPK activation, leading to HIF‐1α degradation via the ubiquitin‐proteasome system. Aligned with these findings, we speculate that within a high‐oxygen environment, TSPCs experience altered intracellular HIF‐1α expression and undergo degradation through the ubiquitin‐proteasome system.

Moreover, in this study, excessive ROS promotes the expression of osteogenic‐related genes and proteins of TSPCs, and the reduced HIF‐1α expression enhanced osteogenic differentiation of TSPCs. Li et al.^56^ also found that excessive ROS can facilitate osteogenic differentiation of TSPCs. Furthermore, Yin et al.^31^ indicated that miR‐135‐5p enhances the osteogenic differentiation of MC3T3‐E1 cells by targeting and regulating HIF‐1α. These findings demonstrated that the key role of the excessive expression of ROS and the reduced expression of HIF‐1α in the osteogenic differentiation of TSPCs. Additionally, Kim et al.^33^ proposed that ROS can directly modulate HIF‐1α and identified specific participants such as nitric oxide (NO), certain microRNAs, as well as transcription and post‐translational modifications that mediate the regulatory impact of ROS on HIF‐1α. In our study, we also observed that excessive ROS inhibited HIF‐1α expression within TSPCs, and inhibiting the ROS/HIF‐1α signalling axis significantly attenuated the osteogenic differentiation of TSPCs. Collectively, these studies further underline the crucial role of the ROS/HIF‐1α signalling axis in the osteogenic differentiation of TSPCs, indicating that high oxygen regulates the osteogenic differentiation of TSPCs through the ROS/HIF‐1α signalling axis. In contrast, Balogh et al.^35^ found that HIF‐1α upregulation can lead to osteochondrogenic differentiation of VSMCs, ultimately resulting in vascular calcification. This contrasts with our findings, and there may be several reasons for the inconsistency. Firstly, it may be due to the different cell types and origins. VSMCs are derived from vascular tissue, while TSPCs are derived from tendon tissue. Different types of cells may have different differentiation effects under the action of HIF‐1A. Second, the original environment in which the two kinds of cells are different. VSMCs are derived from blood vessels and generally exists in hyperoxic environment, while TSPCs are derived from tendons, which are hypovascular tissues, and TSPCs generally exist in hypoxic environment. Hypoxia changes the original environment of VSMCs, and the up‐regulation of HIF‐1A may cause the differentiation of VSMC towards osteogenesis. But, hypoxia is the primary environment of TSPCs, and HIF‐1A is beneficial to maintain the tenogenic differentiation phenotype and inhibit osteogenic differentiation in TSPCs.

NAC is a commonly used antioxidant that mitigates the effects of high oxygen levels by inhibiting and clearing the production of free radicals, while also regulating the metabolic activity of cells.^57^, ^58^ Additionally, research has indicated a proactive role of NAC in preventing and treating tissue calcification. The results of this study revealed that NAC significantly suppressed the osteogenic differentiation of TSPCs. Local injection of NAC markedly inhibited calcification in the Achilles tendon tissues of diabetic rats in vivo. Levy et al.^59^ reported that NAC notably inhibited cardiac valve calcification in rats. Ye et al.^60^ discovered that NAC reduced intracellular glutathione (GSH) levels, ultimately restraining VSMC calcification. Collectively, these findings indicate the positive role of NAC in preventing and treating diabetic calcific tendon disease. This is likely attributed to the reduction of intracellular ROS content of NAC within TSPCs, alleviating oxidative stress responses and inhibiting the ROS/HIF‐1α signalling axis. This mitigates osteogenic differentiation and tissue calcification of TSPCs. These results further underscore the role of high oxygen in regulating the osteogenic differentiation of TSPCs through the ROS/HIF‐1α signalling axis.

This study is the first to investigate the origins and associated molecular mechanisms underlying tendon calcification in patients with diabetes by focusing on local tissue oxygen concentrations. Through experiments involving the use of the NAC in vitro and in vivo, we validated the potential of NAC to inhibit osteogenic differentiation and tissue calcification of TSPCs. This innovative approach provides novel insights and strategies for the prevention and treatment of diabetic calcific tendon diseases. Nevertheless, our study has some limitations. First, we haven't got enough human diabetic and non‐diabetic tendon tissue to test it. An important reason is the limited supply of samples and ethical constraints. We plan to continue to invest more effort and resources in collecting relevant specimens, and to collaborate with other research institutions, medical institutions, or patients to obtain more tendon tissue samples. We believe that as our work continues, more data will be available for analysis in the future. Furthermore, we are currently unable to directly quantify oxygen concentrations in living tissues. This has certain restrictions on our deep understanding of the functions and activities of biological organizations. At present, we are exploring a strategy to estimate oxygen levels in tendon tissue by analysing blood vessels and haemoglobin content in the tissue. Blood vessels are channels for blood, and haemoglobin is the main carrier of oxygen. Therefore, theoretically, blood vessel and haemoglobin content have an important correlation with oxygen concentration in tissues. Although this method estimates oxygen concentrations indirectly, under current conditions we can only look for this alternative. And through as accurate as possible speculation, for follow‐up research to provide the basis. But that doesn't mean we'll always be bound by these technological limitations. Our team is working to explore new ways to address this limitation and refine the experimental data.

## 5 CONCLUSIONS

Our study confirmed the presence of increased blood vessels, haemoglobin levels, and a hyperoxic environment in diabetic tendon tissues. Hyperoxia promotes the osteogenic differentiation of TSPCs through the ROS/HIF‐1a signalling axis, ultimately leading to tendon tissue calcification. This study provides insights into the pathogenesis of diabetic calcific tendinopathy and a new target for preventing and treating this disease, which has important clinical significance.

## AUTHOR CONTRIBUTIONS

**Ming Zhang:** Data curation (lead); validation (lead); writing – original draft (lead). **Guan‐Chun Dai:** Data curation (equal); formal analysis (equal); writing – review and editing (equal). **Yuan‐Wei Zhang:** Formal analysis (equal); methodology (equal); software (equal). **Pan‐Pan Lu:** Data curation (equal); formal analysis (equal); methodology (equal). **Hao Wang:** Formal analysis (equal); methodology (equal); software (equal). **Ying‐Juan Li:** Formal analysis (equal); methodology (equal); software (equal). **Yun‐Feng Rui:** Conceptualization (lead); funding acquisition (lead); writing – review and editing (lead).

## FUNDING INFORMATION

This study was supported by the National Natural Science Foundation of China (No.81871812); Natural Science Foundation of Jiangsu Province (BK20221462). Research Personnel Cultivation Programme of Zhongda Hospital Southeast University (CZXM‐GSP‐RC46); Winfast Charity Foundation Project (YL20220525).

## CONFLICT OF INTEREST STATEMENT

The authors declare no conflict of interest. This article has already been uploaded to a preprint. (https://www.researchsquare.com/article/rs‐3417115/v1).

## Supporting information


File S1.

File S2.

## Data Availability

The data and materials of the study can be obtained from the corresponding author upon request.
