# Supplementary material for: Enhancing osteogenic differentiation of diabetic tendon stem/progenitor cells through hyperoxia: Unveiling ROS/HIF‐1α signalling axis
Source: J Cell Mol Med. 2024 Oct 28;28(20):e70127. doi: 10.1111/jcmm.70127 (PMC11518821; doi:10.1111/jcmm.70127)
Supplement: Supplementary file 1 — File S1. [file JCMM-28-e70127-s001.docx]

**Supplementary materials**

**Figure S1.** Verification of the self-renewal capacity and multipotent differentiation potential of the primary cells. (A-D) Crystal Violet Staining. (E)ARS staining. (F) Alcian blue staining. (F) Oil Red O staining.

**Figure S2.** TSPCs were treated with high glucose intervention in healthy rats for 1 week. (A) qRT-PCR was performed to detect VEGF mRNA expression in each group. (B–C) WB was used to detect the protein expression of VEGF in each group. Consequently, a semi-quantitative analysis of WB results was performed. All full‑length blots are presented in Additional file 2: Figure S2B. (D–E) IF was used to detect the protein expression of VEGF. Semi-quantitative analysis of IF in each group was performed. Data are presented as the mean ± standard deviation. n = 3 for each group, **P＜0.01, ***P＜0.001, ****P＜0.0001. Scale bar: 50 μm.

LO-LG: Low oxygen and low glucose, LO-HG: Low oxygen and high glucose, TSPCs: Tendon Stem/Progenitor Cells, qRT-PCR: Quantitative real-time Polymerase Chain Reaction, VEGF: Vascular Endothelial Growth Factor, mRNA: message RNA , WB: Western Blot

**Figure S3.** TSPCs isolated from healthy rats were cultured with LO-LG, LO-HG, and HO-HG for 2 weeks. (A–B) The protein expression of RUNX-2 in TSPCs was detected by IF, and a semi-quantitative analysis of IF results was performed. (C–D) OCN protein expression in TSPCs was detected by IF. Semi-quantitative analysis of IF was performed. Data are presented as the mean ± standard deviation. n = 3 for each group, **P＜0.01, ***P＜0.001, ****P＜0.0001. Scale bar: 50 μm.

LO-LG: Low oxygen and low glucose, LO-HG: Low oxygen and high glucose, HO-HG: High oxygen and high glucose, TSPCs: Tendon Stem/Progenitor Cells, IF: Immunofluorescence

**Figure S4.** Hyperoxia inhibited tenogenic differentiation of TSPCs. (A–E) TSPCs isolated from healthy rats were cultured with LO-LG, LO-HG, and HO-HG for 2 weeks. mRNA expression levels of MKX, SCX, TNC, Tnmd, and Col-3 in TSPCs were detected using qRT-PCR. (F) The protein expression levels of SCX and Col-3 in TSPCs were detected by WB. All full‑length blots are presented in Additional file 2: Figure S3F. (G–H) Semi-quantitative WB analysis was performed. Data are presented as the mean ± standard deviation. n = 3 for each group, *P＜0.05, **P＜0.01, ***P＜0.001, ****P＜0.0001.

LO-LG: Low oxygen and low glucose, LO-HG: Low oxygen and high glucose, HO-HG: High oxygen and high glucose, TSPCs: Tendon Stem/Progenitor Cells, qRT-PCR: Quantitative real-time Polymerase Chain Reaction, WB: Western Blot

**Figure S5.** Hyperoxia-induced changes in mitochondrial morphology, cellular oxidation, and antioxidant capacity of TSPCs. (A–C) TSPCs isolated from healthy rats were cultured with LO-LG, LO-HG, and HO-HG for 3 weeks, and the morphological and structural changes in mitochondria were observed by TEM. (D–E) TSPCs isolated from healthy rats were cultured with LO-LG, LO-HG, and HO-HG for 1 week, and lipid oxidation and total antioxidant capacity of TSPCs were observed. Data are presented as the mean ± standard deviation. n = 3 for each group, *P＜0.05, **P＜0.01, ****P＜0.0001. Scale bar: 500 nm.

LO-LG: Low oxygen and low glucose, LO-HG: Low oxygen and high glucose, HO-HG: High oxygen and high glucose, TSPCs: Tendon Stem/Progenitor Cells

**Figure S6.** ROS regulated the expression of HIF-1a. (A)TSPCs were cultured with LO-HG, HO-HG, and LO-HG+H_2_O_2_ for 3 days. The mRNA expression level of HIF-1a in TSPCs was detected by qRT-PCR. (B–C). The protein expression level of HIF-1a in TSPCs was detected by WB, and a semi-quantitative analysis of WB results was performed. All full‑length blots are presented in Additional file 2: Figure S5B. (D–E). The protein expression level of HIF-1a in TSPCs was detected by IF, and a semi-quantitative analysis of IF results was performed. Data are presented as the mean ± standard deviation. n = 3 for each group, ns: not significant, *P＜0.05, ****P＜0.0001.

LO-HG: Low oxygen and high glucose, HO-HG: High oxygen and high glucose, H_2_O_2_: Hydrogen Peroxide, TSPCs: Tendon Stem/Progenitor Cells, qRT-PCR: Quantitative real-time Polymerase Chain Reaction, WB: Western Blot, IF: Immunofluorescence

**Supplementary Table S1.** Primer sequences used in the experiment.

**Supplementary Table S2.** Antibody information relevant to the experiment.

**Figure S1**


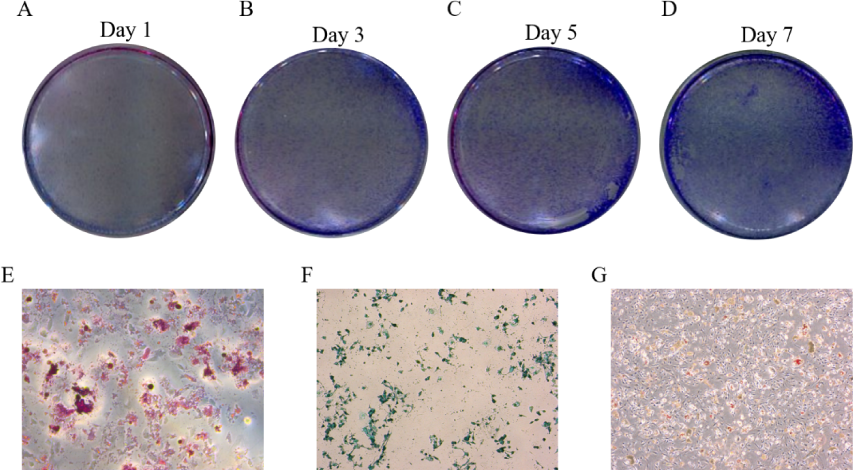


**Figure S2**


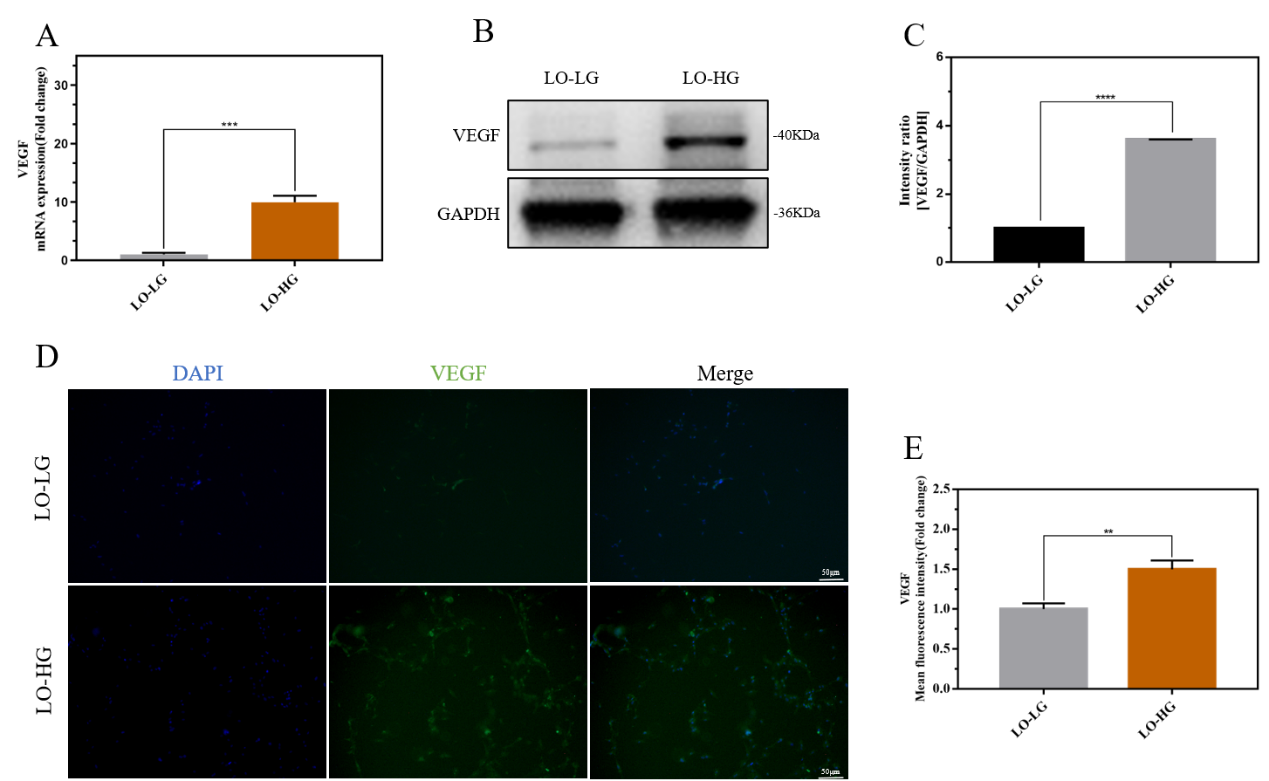


**Figure S3**

**
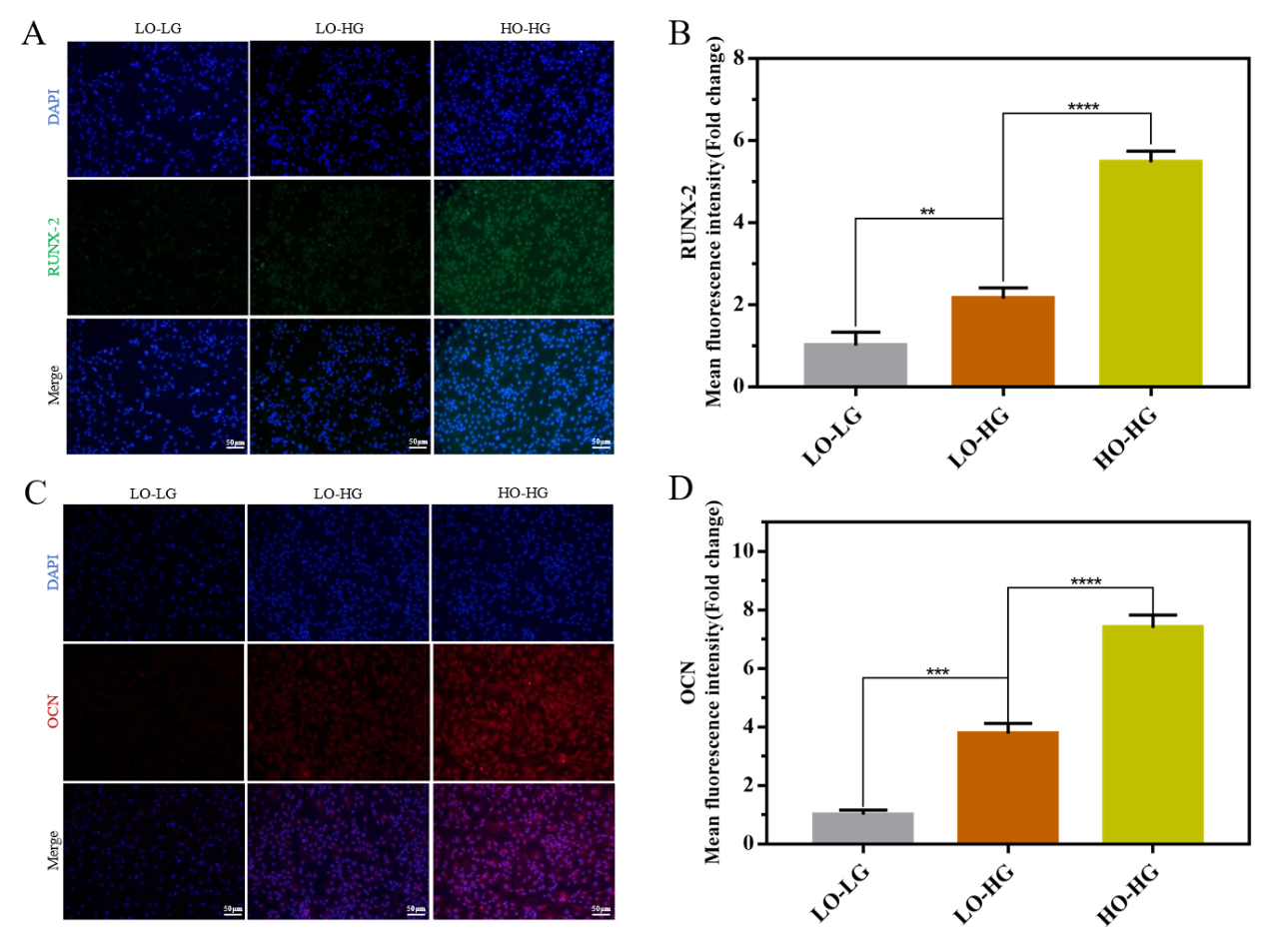
**

**Figure S4**

**
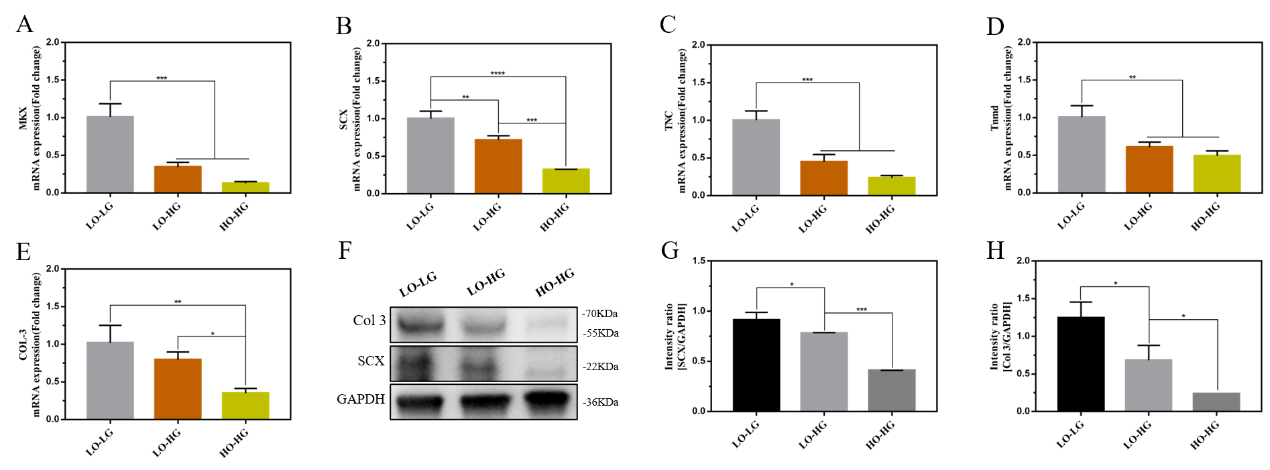
**

**Figure S5**

**
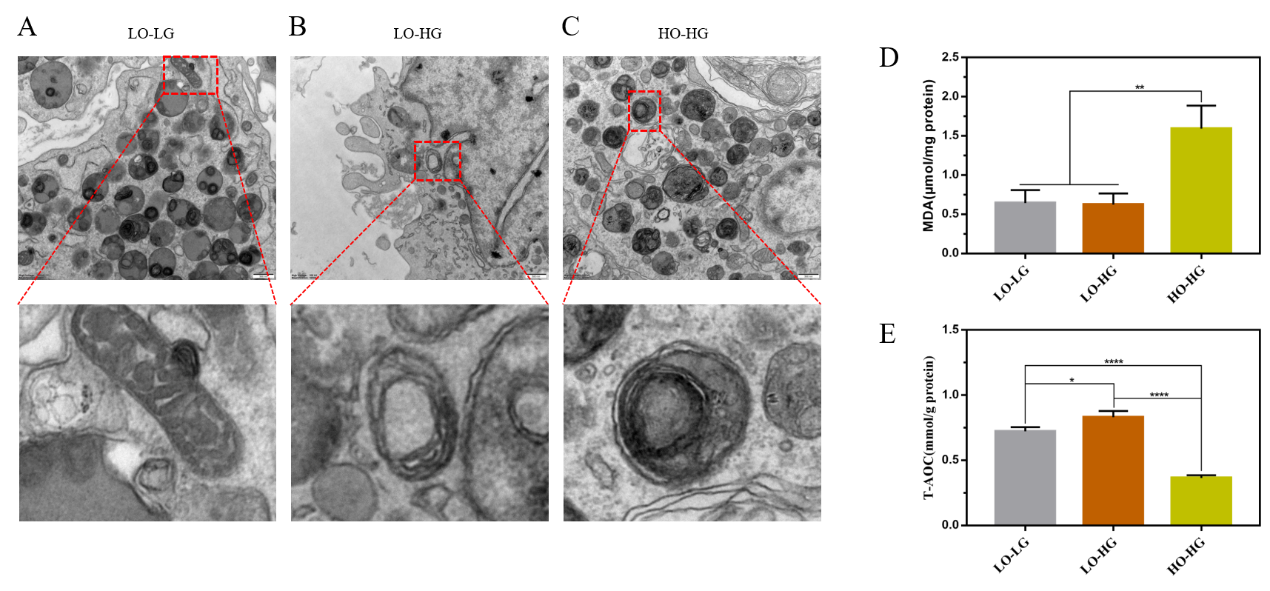
**

**Figure S6**

**
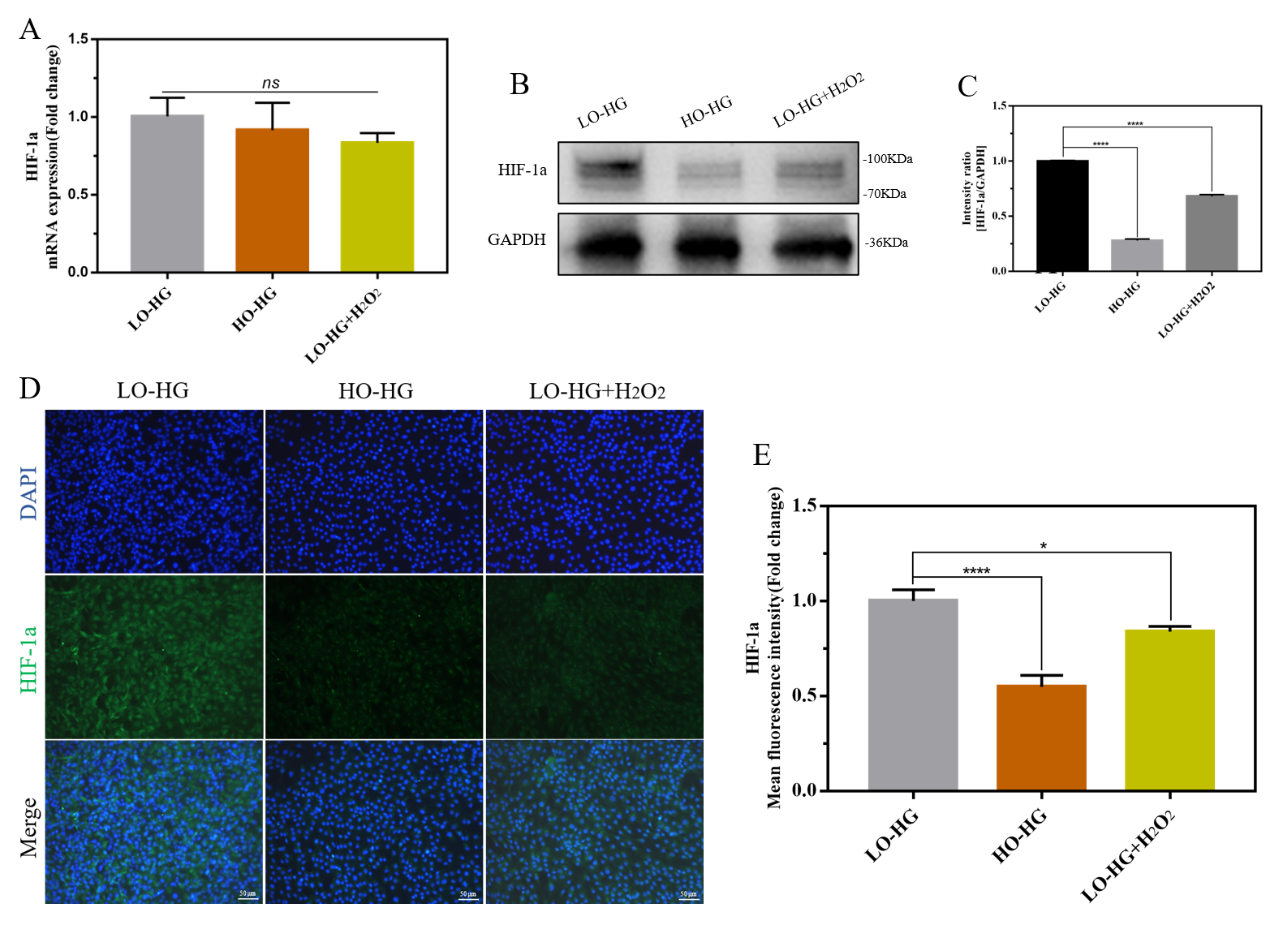
**

**Table S1**

| Genes | 5′-3′ | Sequence |
| --- | --- | --- |
| GAPDH(Rat) | Forward  Reverse | CGCCTGGAGAAAGCTGCTAA  CGACCTGGTCCTCGGTGTAG |
| VEGF(Rat) | Forward  Reverse | TCCCTCAGCCTACCATCAAGT  CACCAATGTGCTAACCGTCTT |
| ALP(Rat) | Forward  Reverse | CGGCGGATGATAAGGAGGACT  CGTGTAACAGATGGAAACCGTAGA |
| RUNX-2(Rat) | Forward  Reverse | AACCAAGAAGGCACAGACAGA  GGCTCAGATAAGAGGGGTAAGA |
| OCN(Rat) | Forward  Reverse | GCTCACTCTGCTGGCCCTGAC  CCTTACTGCCCTCCTGCTTGG |
| OPN(Rat) | Forward  Reverse | ATCCAAGGATGCCAACGACTC  AGCCATTGACCACCAAGAAGC |
| MKX(Rat) | Forward  Reverse | GCCATCGTAGGACACTTTCTC  TCATCCACTCATACAGTCAGCA |
| SCX(Rat) | Forward  Reverse | CAACGTGCTACTGGTGGGTGA  CTGTTTGGGCTGGGTGTTCTC |
| TNC(Rat) | Forward  Reverse | GACCTTGATTTCTCCGACCTG  ACCACTGGCTGATTCTCCTCT |
| Tnmd(Rat) | Forward  Reverse | CAGGACTTTGAGGAGGATGGT  TTGGTAGCAGTAGGGGTATGG |
| Col-3(Rat) | Forward  Reverse | TGCCACCCTGAACTCAAGAGC  AGCACCAGCATCTGTCCACCA |
| HIF-1a(Rat) | Forward  Reverse | AGGATGGAATGGAGCAGAAGA  CAGAAACGAAACCCCACAGAC |

**Table S2**

| Antibody | Host | Source |
| --- | --- | --- |
| GAPDH Monoclonal antibody | Mouse | Proteintech, Wuhan, China |
| Osteopontin Monoclonal antibody | Mouse | Santa Cruz Biotechnology, Shanghai, China |
| Osteonectin Monoclonal antibody | Mouse | Santa Cruz Biotechnology, Shanghai, China |
| ALP Monoclonal antibody | Mouse | Santa Cruz Biotechnology, Shanghai, China |
| VEGFA Monoclonal antibody | Mouse | Proteintech, Wuhan, China |
| RUNX-2 Polyclonal antibody | Rabbit | Proteintech, Wuhan, China |
| Osteocalcin Polyclonal antibody | Rabbit | Proteintech, Wuhan, China |
| Scleraxis Monoclonal antibody | Mouse | Santa Cruz Biotechnology, Shanghai, China |
| Collagen Type III Polyclonal antibody | Rabbit | Proteintech, Wuhan, China |
| HIF-1 alpha Monoclonal antibody | Mouse | Proteintech, Wuhan, China |
| Ubiquitin Polyclonal antibody | Rabbit | Proteintech, Wuhan, China |
| HRP-conjugated Affinipure Goat Anti-Mouse IgG(H+L) | Goat | Proteintech, Wuhan, China |
| HRP-conjugated Affinipure Goat Anti-Rabbit IgG(H+L) | Goat | Proteintech, Wuhan, China |
| ABflo® 488-conjugated Goat Anti-Rabbit IgG (H+L) | Goat | ABclonal Technology, Wuhan, China |
| ABflo® 488-conjugated Goat Anti-Mouse IgG (H+L) | Goat | ABclonal Technology, Wuhan, China |
| ABflo® 594-conjugated Goat Anti-Mouse IgG (H+L) | Goat | ABclonal Technology, Wuhan, China |
| ABflo® 594-conjugated Goat Anti-Rabbit IgG (H+L) | Goat | ABclonal Technology, Wuhan, China |
