# Supplementary material for: Enhancing osteogenic differentiation of diabetic tendon stem/progenitor cells through hyperoxia: Unveiling ROS/HIF‐1α signalling axis
Source: J Cell Mol Med. 2024 Oct 28;28(20):e70127. doi: 10.1111/jcmm.70127 (PMC11518821; doi:10.1111/jcmm.70127)
Supplement: Supplementary file 2 — File S2. [file JCMM-28-e70127-s002.docx]

**Full‑length blots**


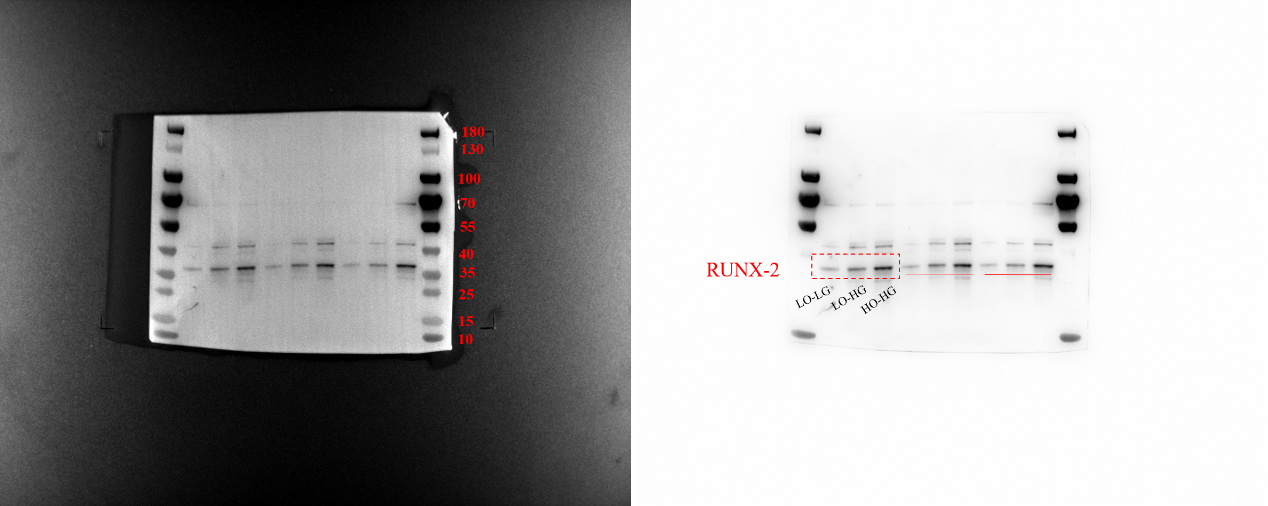


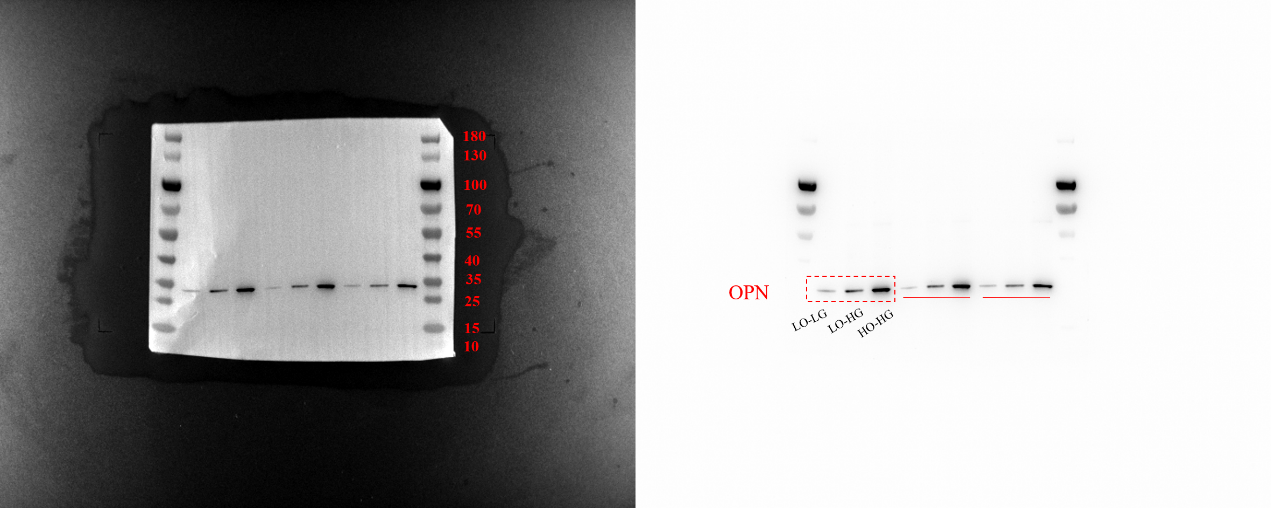


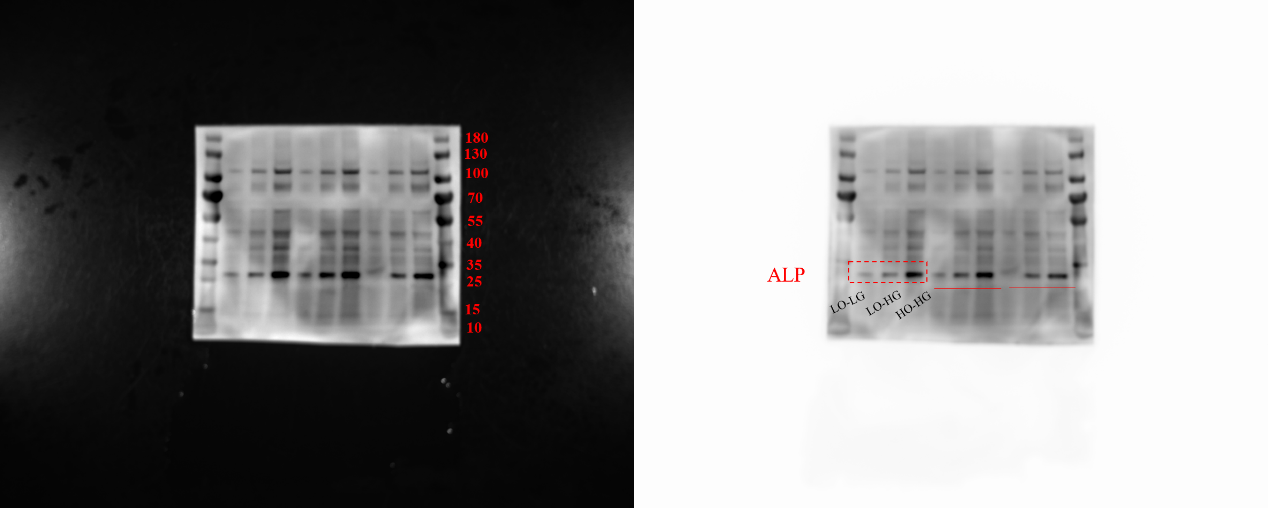


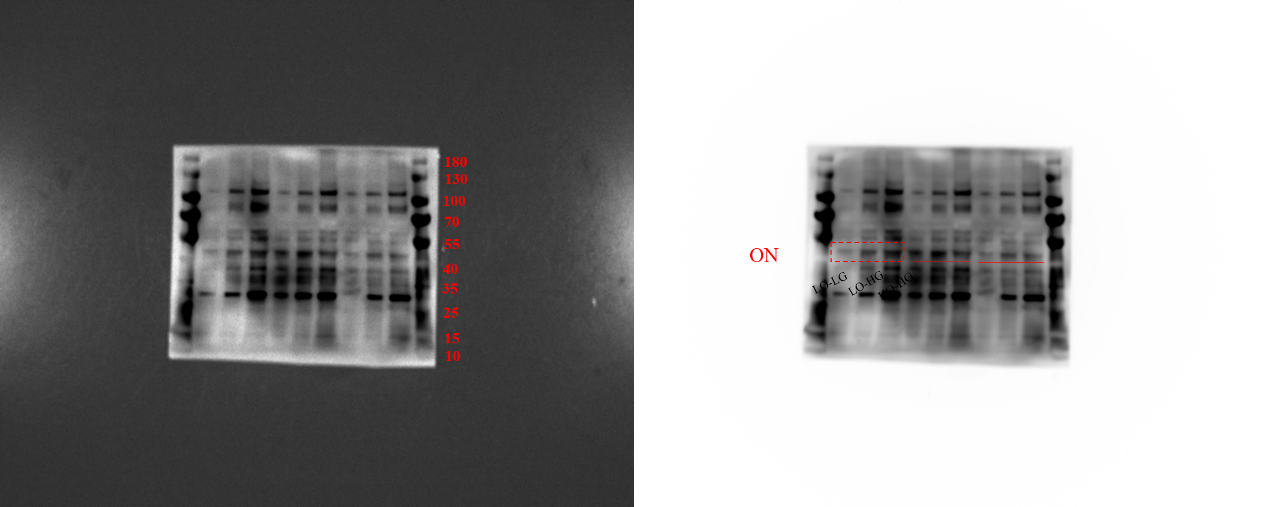


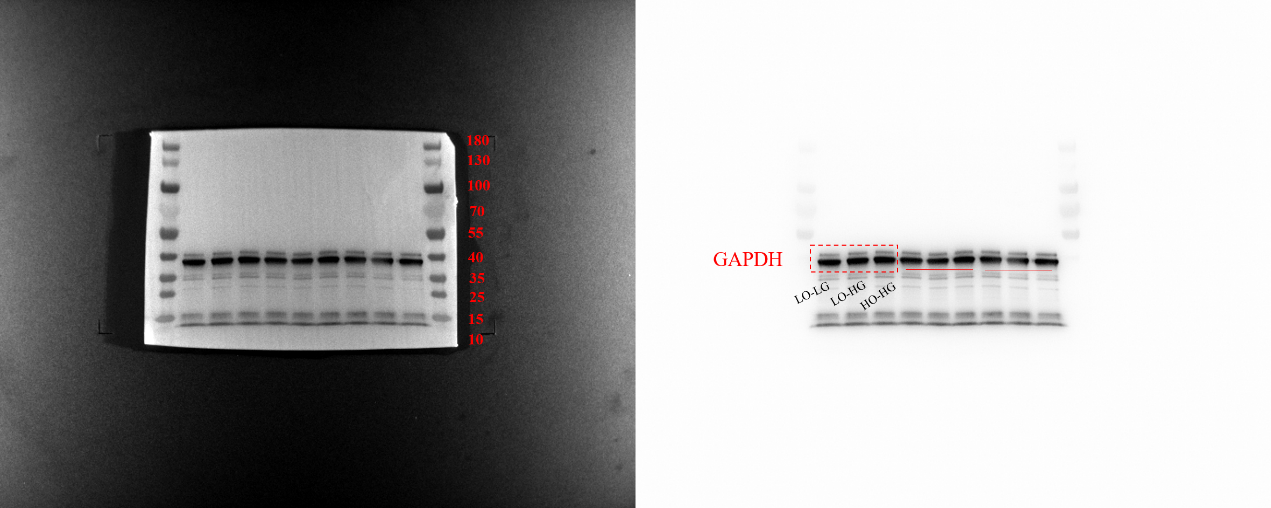


**Figure 3E.** Original western blot gels of RUNX-2 and OPN in the TSPCs. GAPDH was included as a loading control.

**
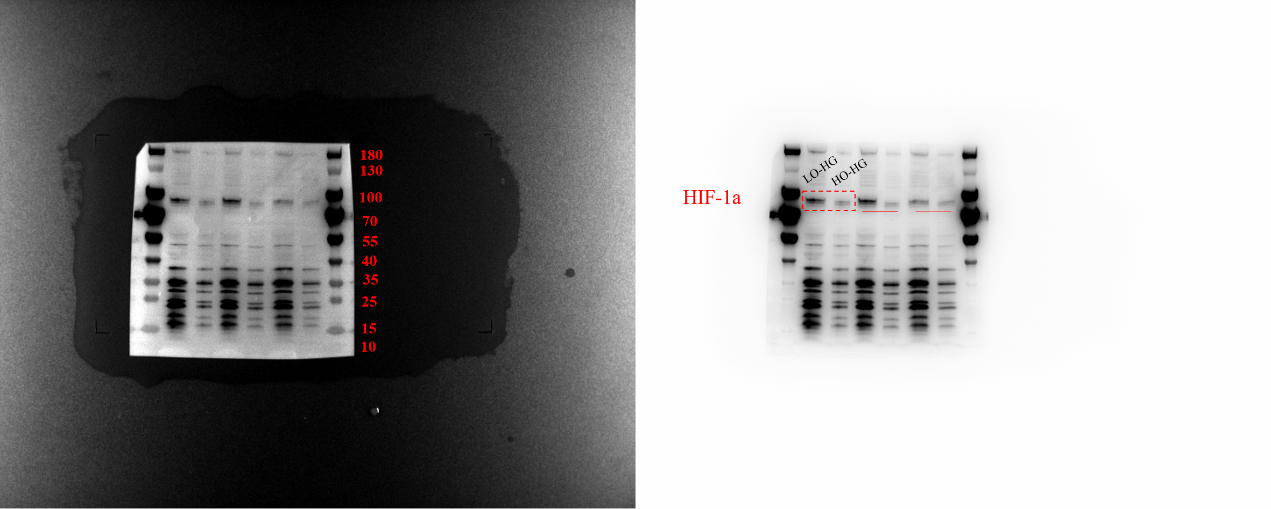
**


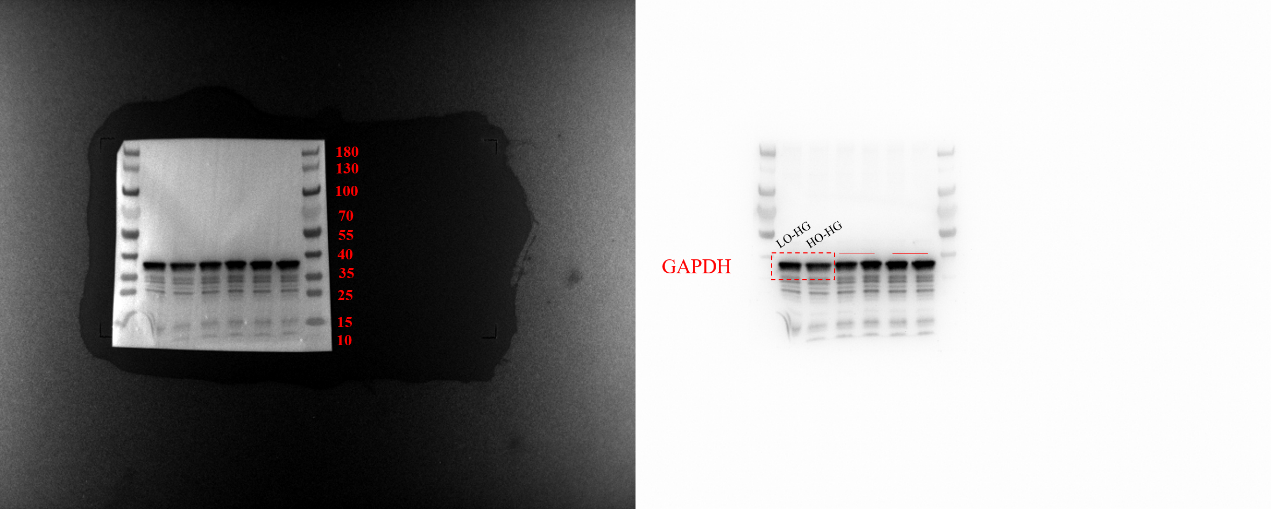


**Figure 4G.** Original western blot gels of HIF-1a in the TSPCs. GAPDH was included as a loading control.

**
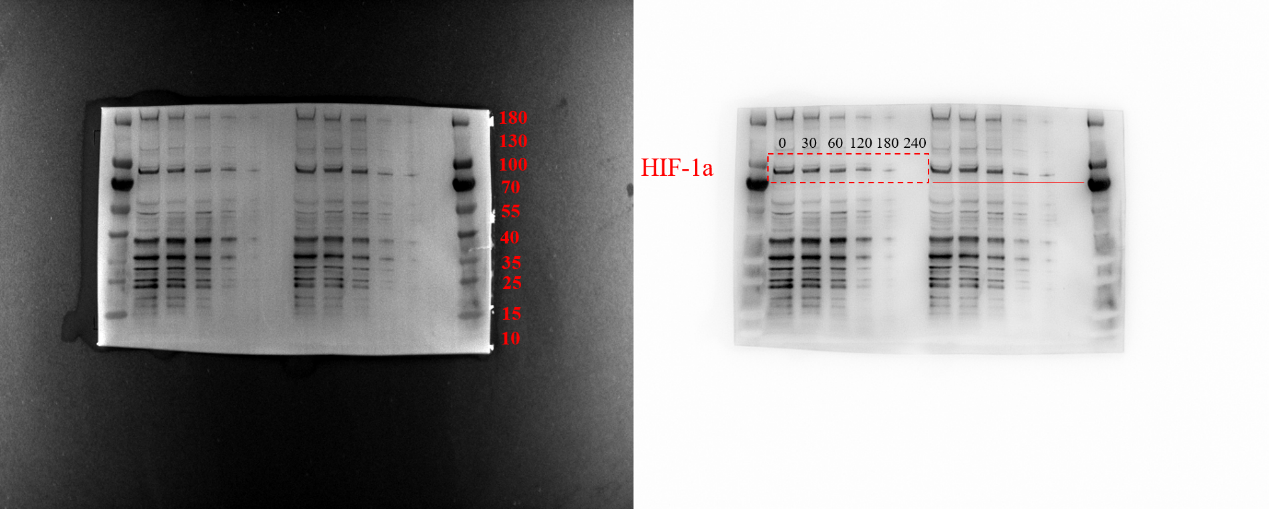
**

**
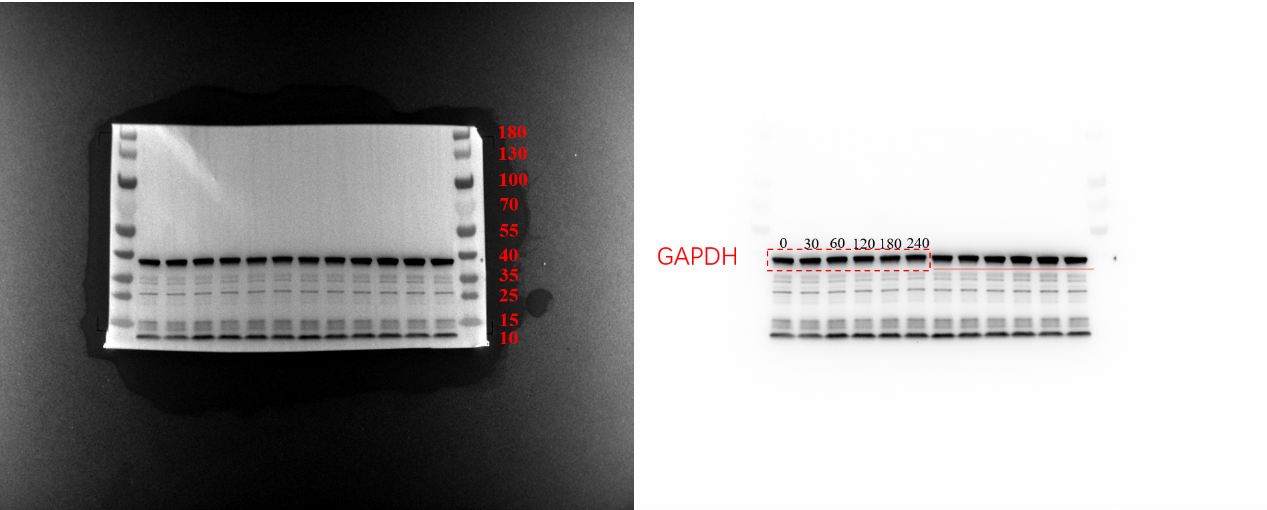
**

**Figure 4I.** Original western blot gels of HIF-1a in the TSPCs. GAPDH was included as a loading control.

**
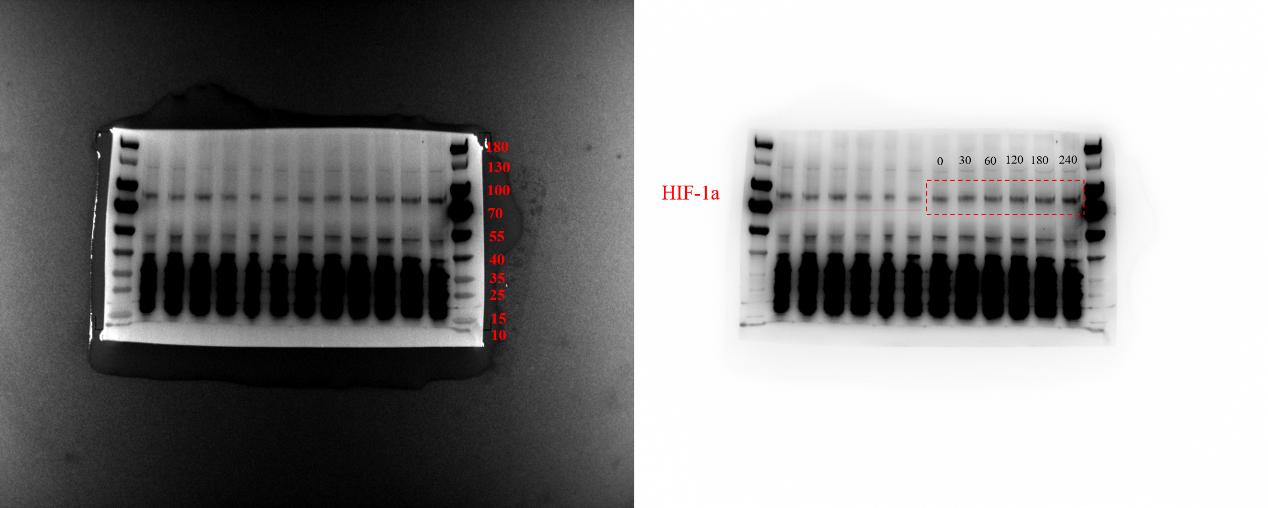

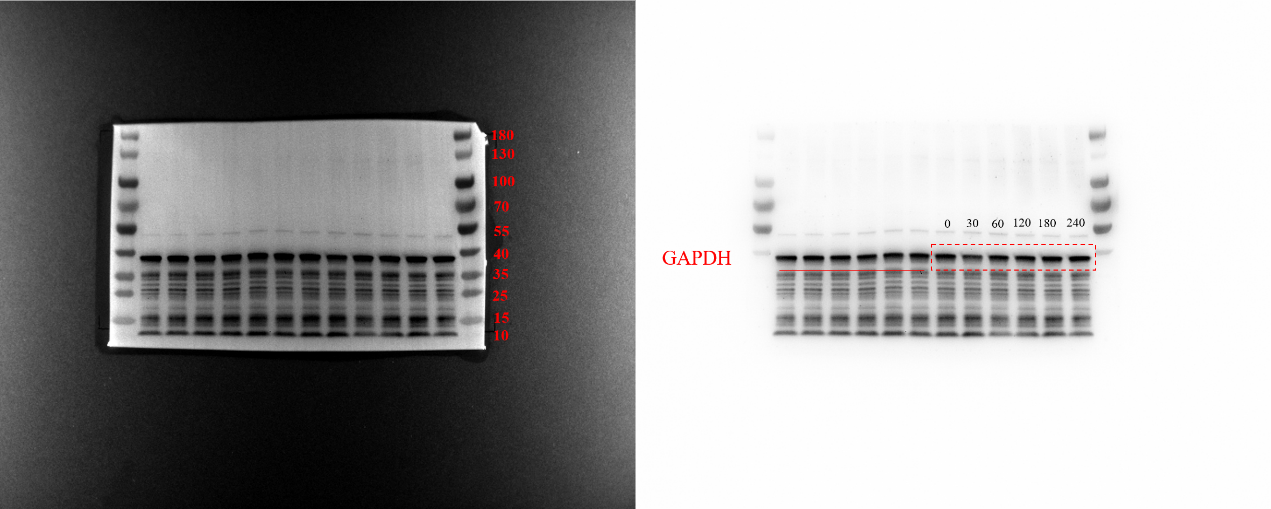
**

**Figure 4J.** Original western blot gels of HIF-1a in the TSPCs. GAPDH was included as a loading control.

**
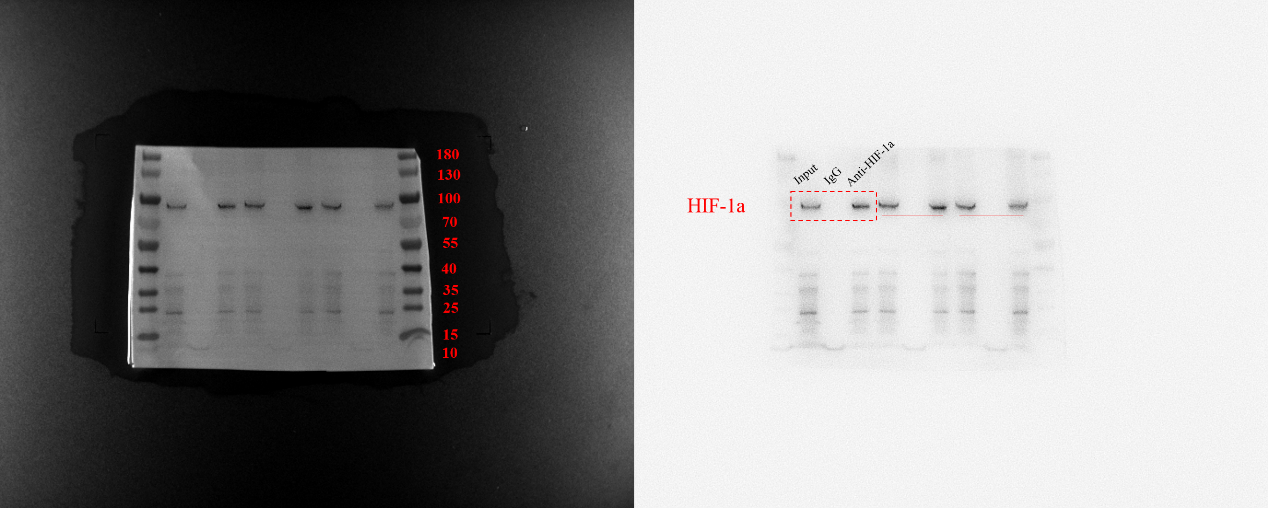

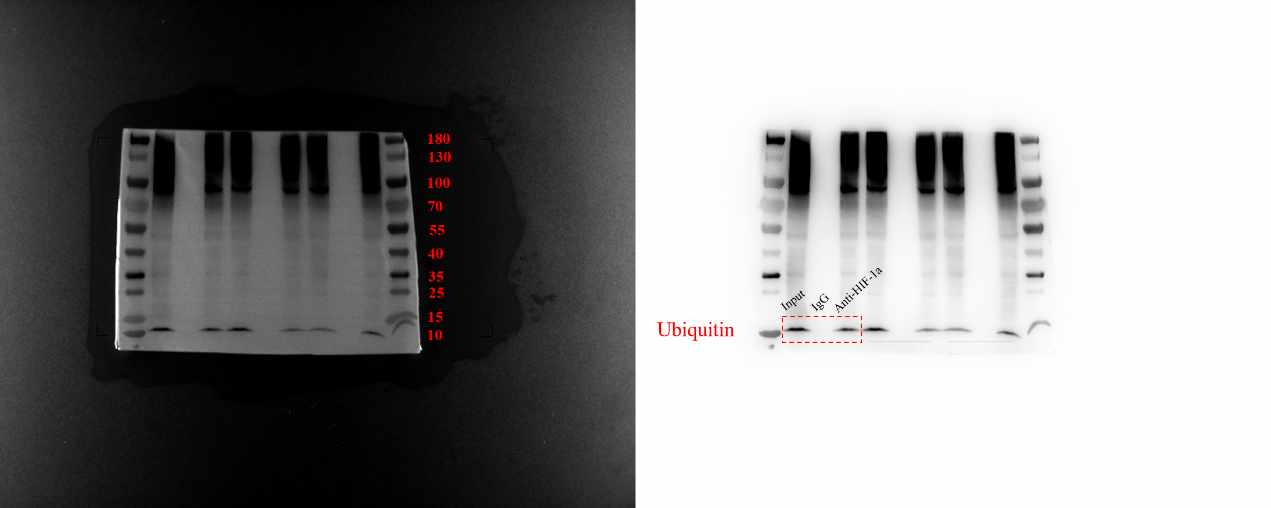
**

**Figure 4K.** Original western blot gels of HIF-1a and Ubiquitin in the TSPCs. GAPDH was included as a loading control.

**
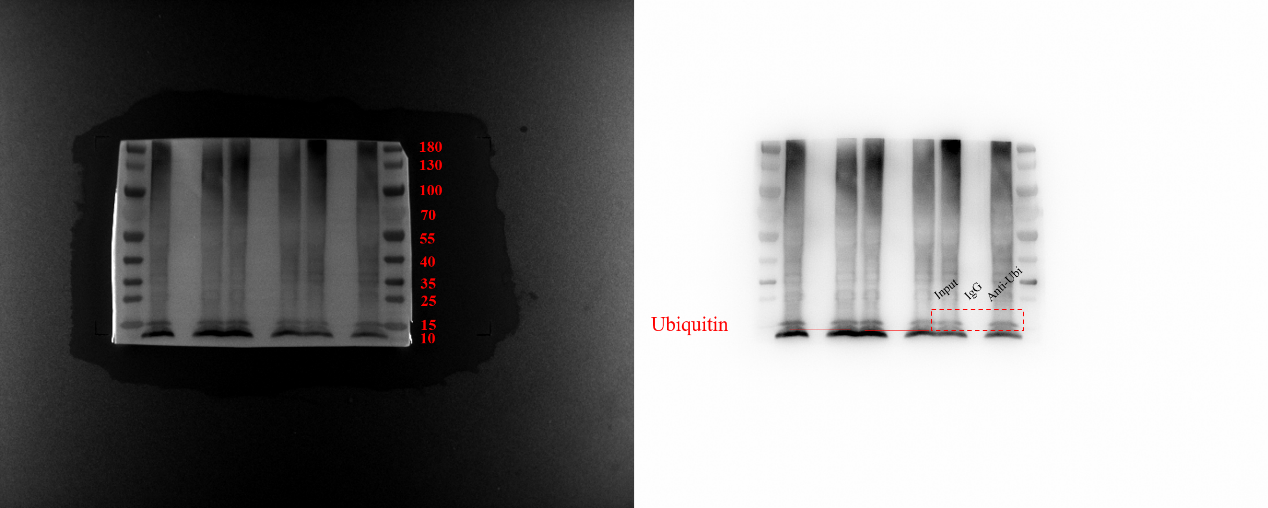

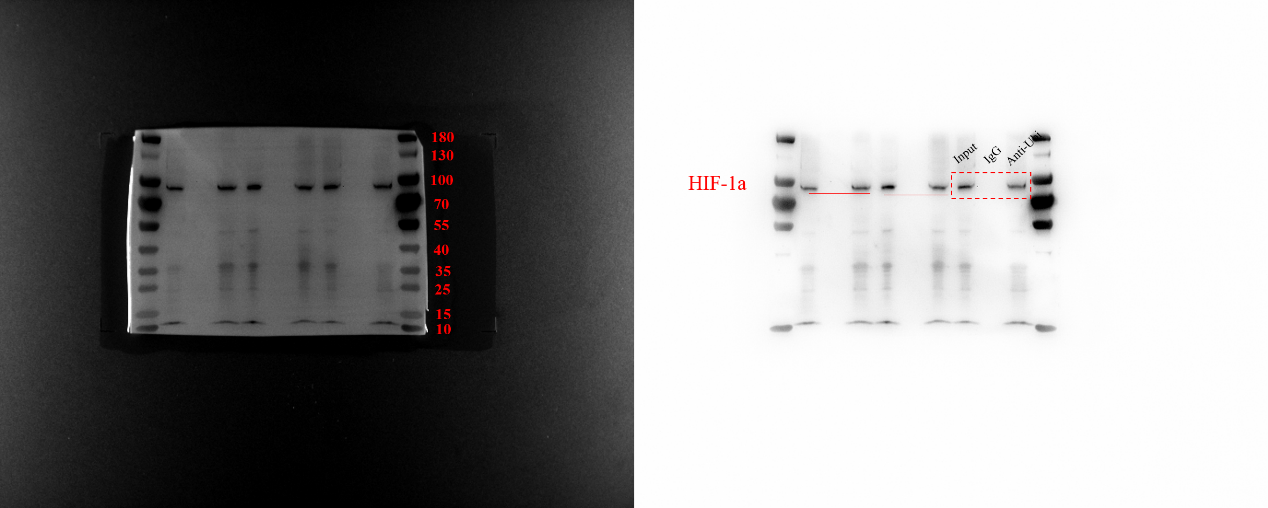
**

**Figure 4L.** Original western blot gels of HIF-1a and Ubiquitin in the TSPCs. GAPDH was included as a loading control.

**
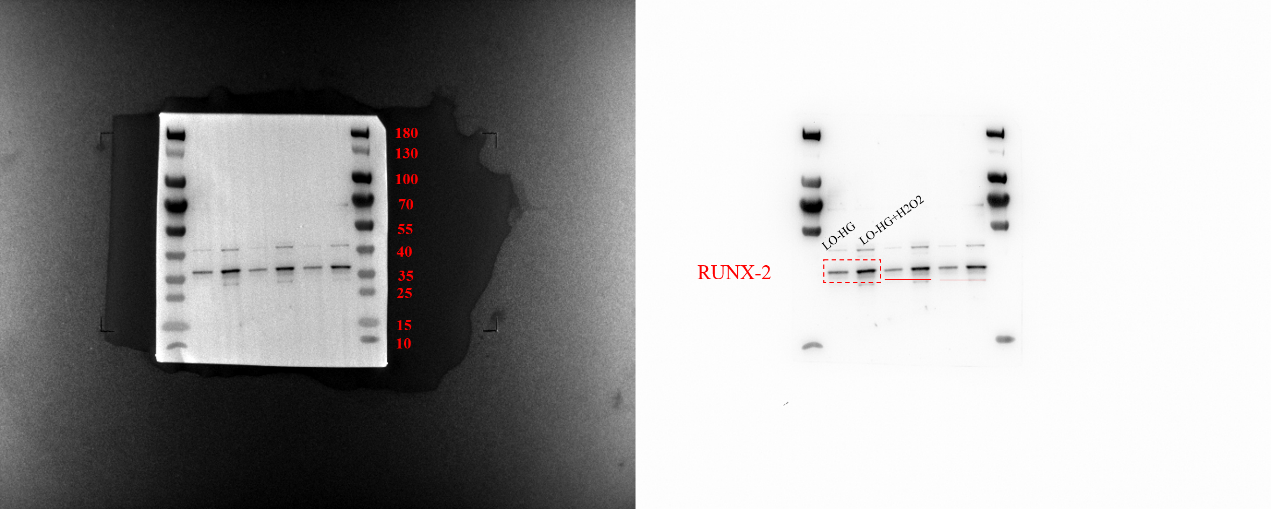

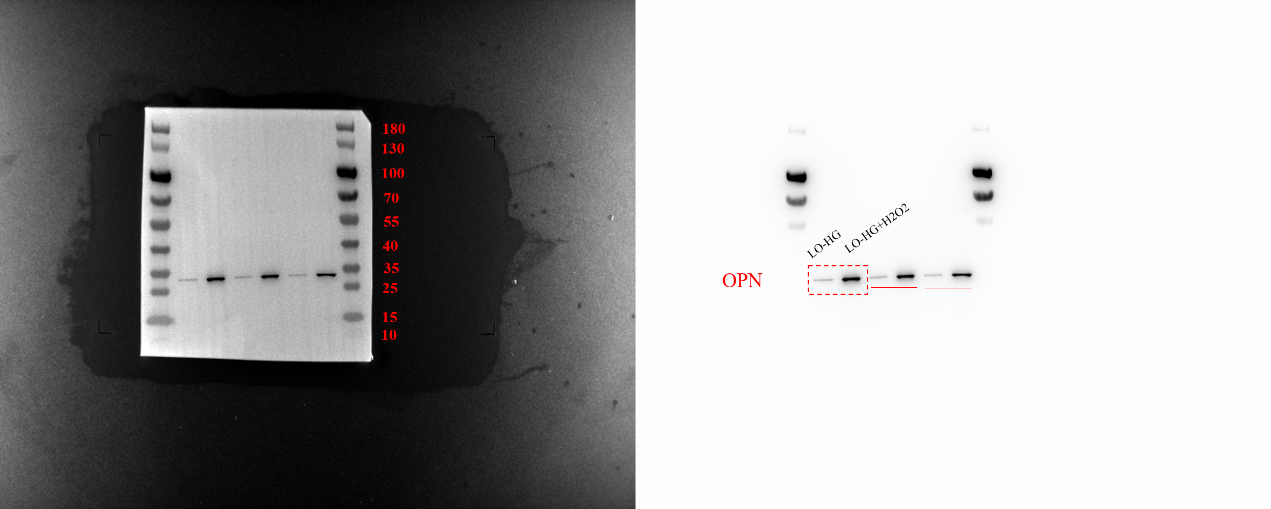

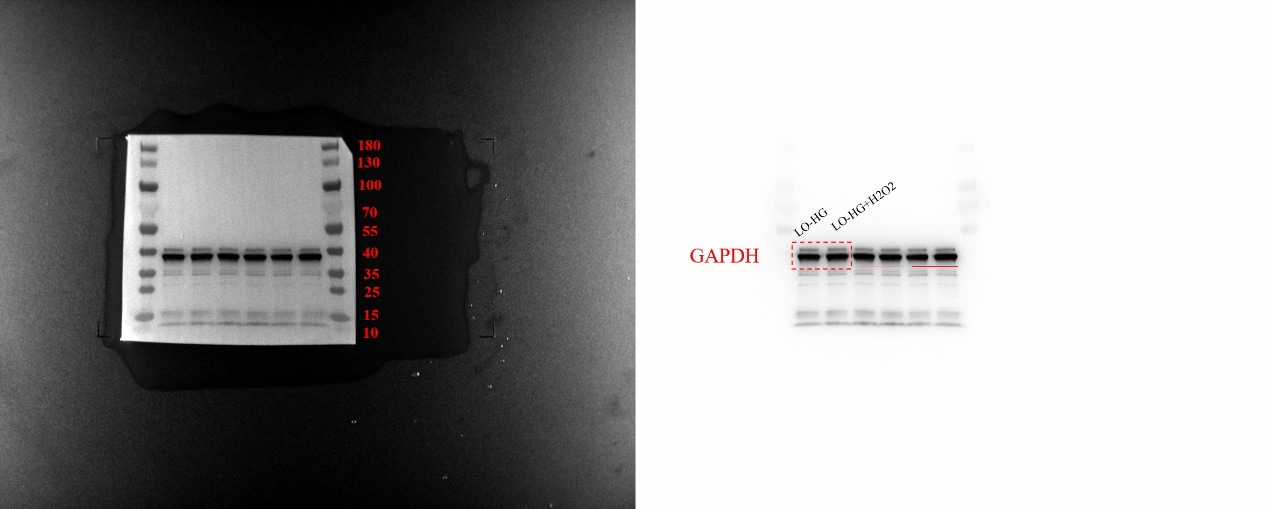
**

**Figure 5E.** Original western blot gels of RUNX-2 and OPN in the TSPCs. GAPDH was included as a loading control.

**
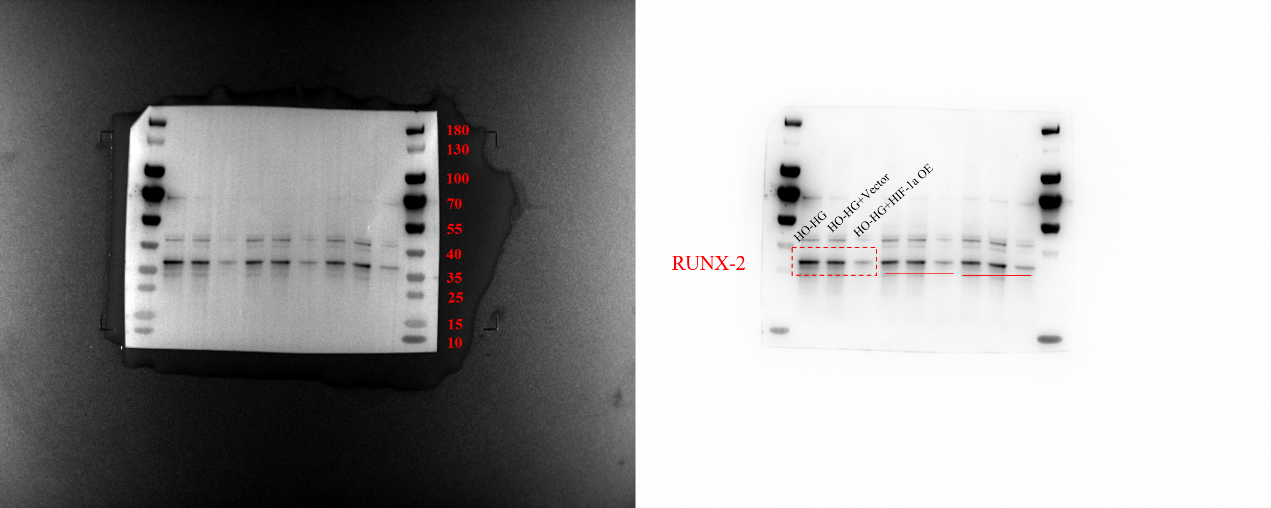

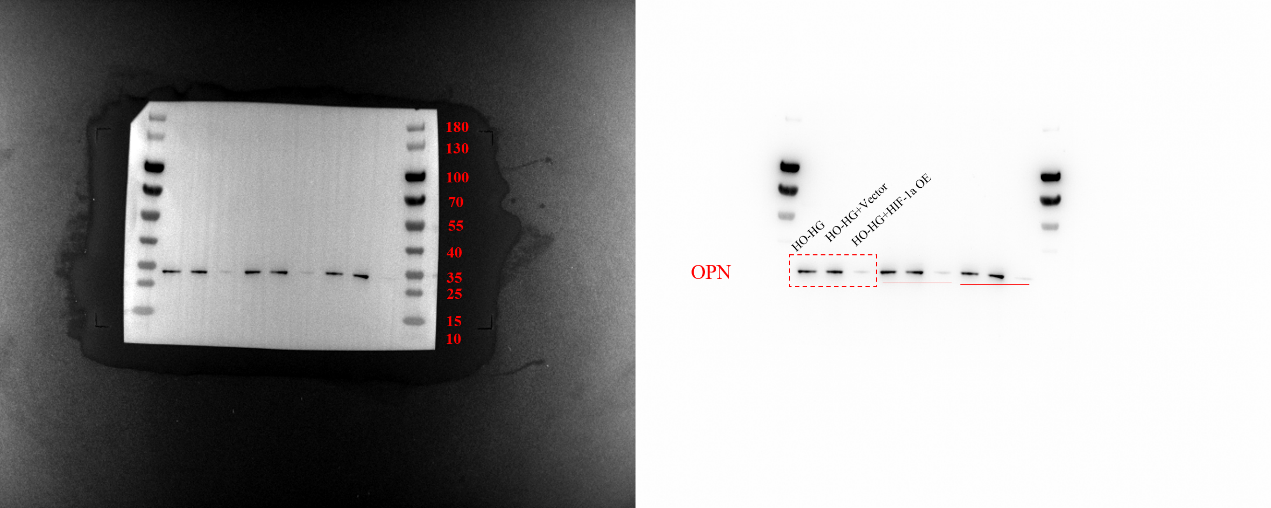

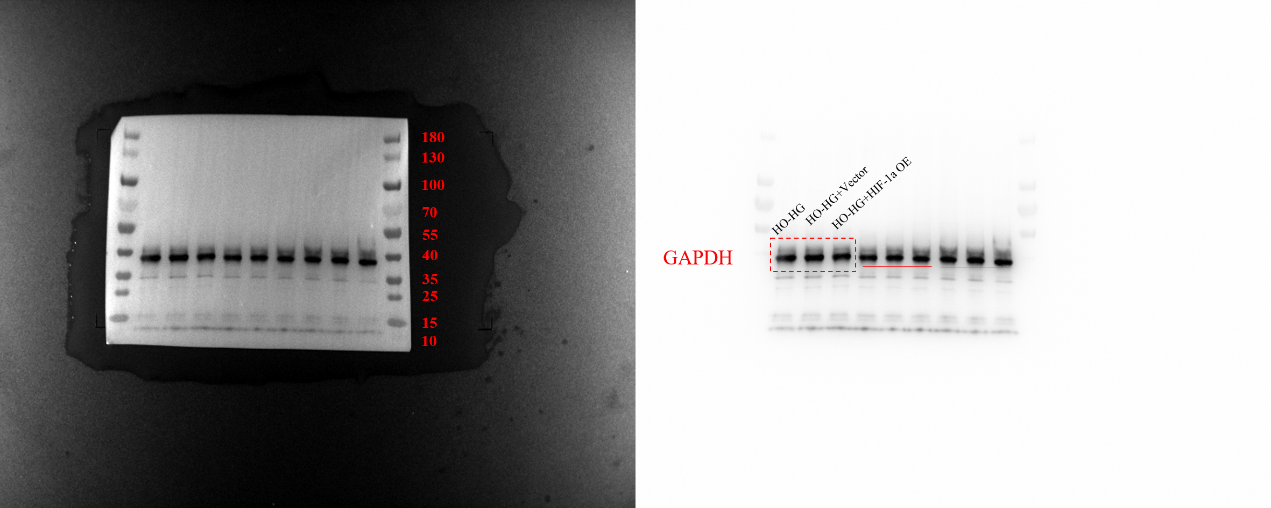
**

**Figure 5M.** Original western blot gels of RUNX-2 and OPN in the TSPCs. GAPDH was included as a loading control.

**
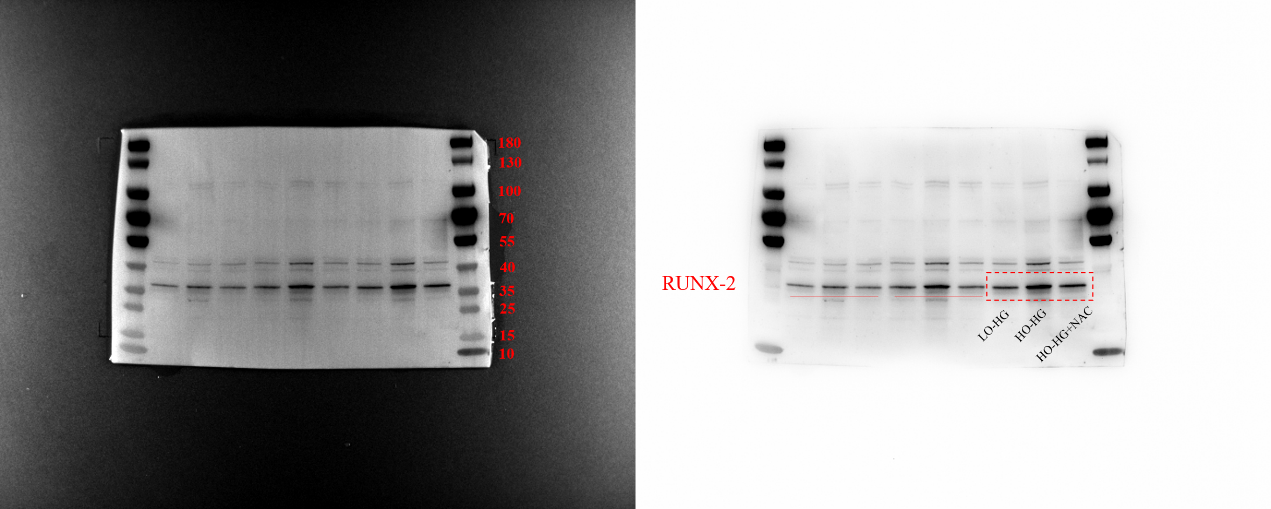

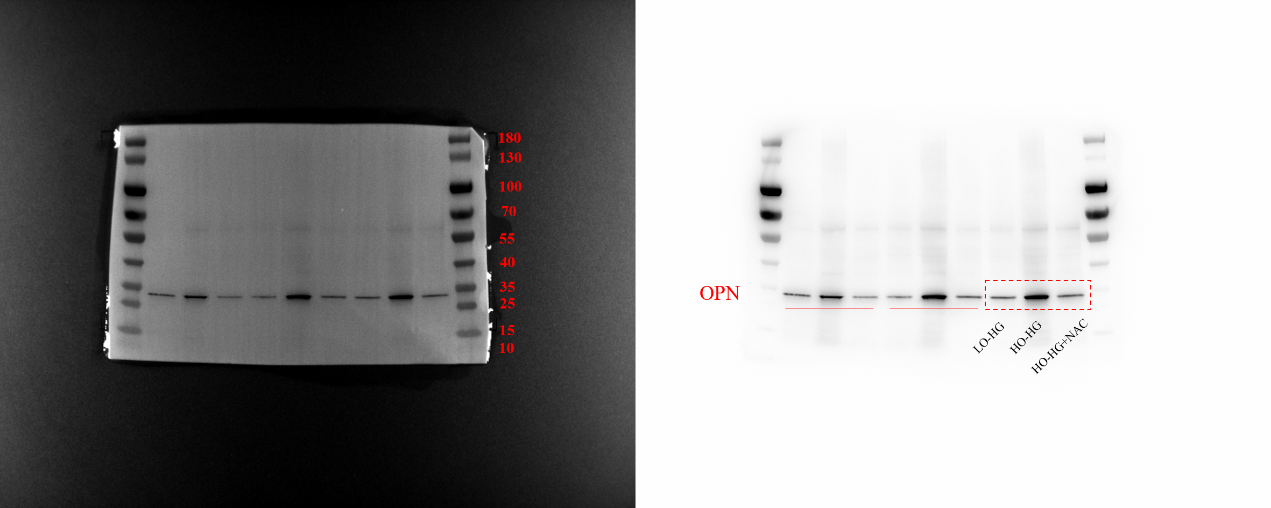

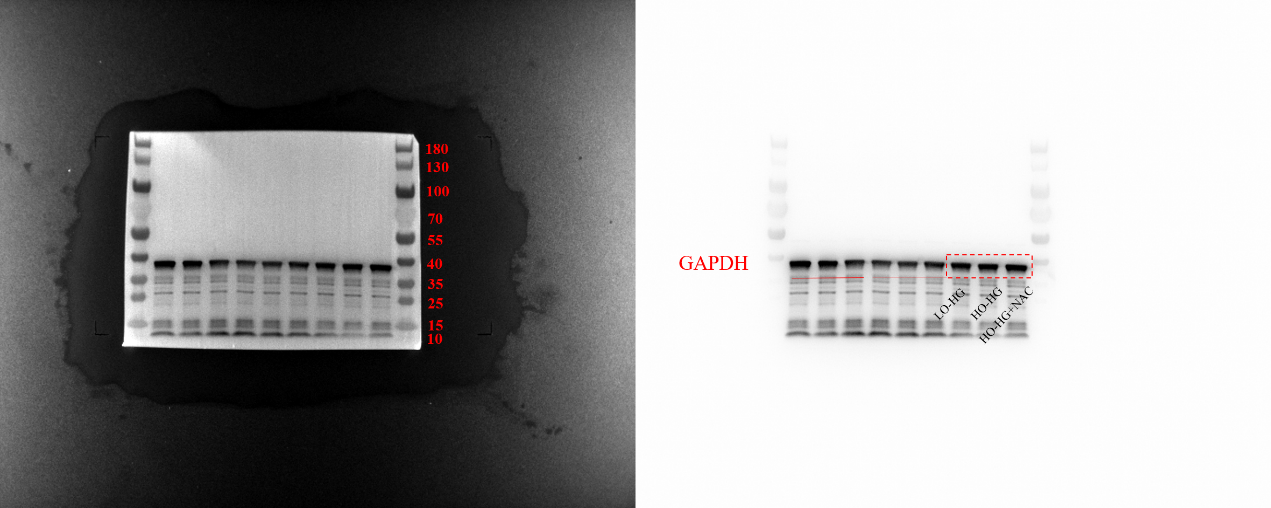
**

**Figure 6E.** Original western blot gels of RUNX-2 and OPN in the TSPCs. GAPDH was included as a loading control.

**
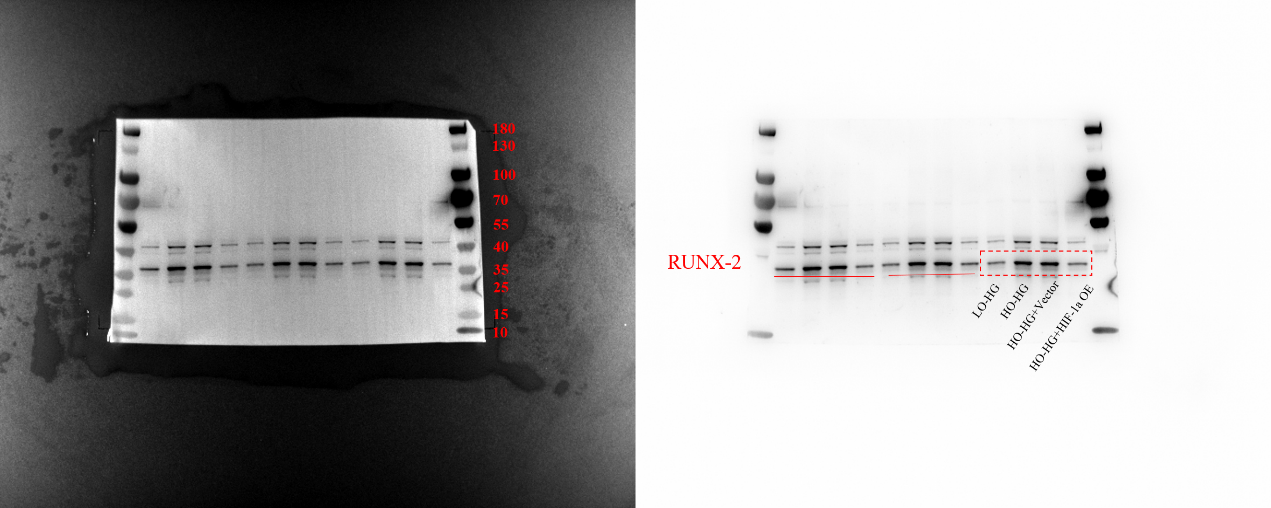

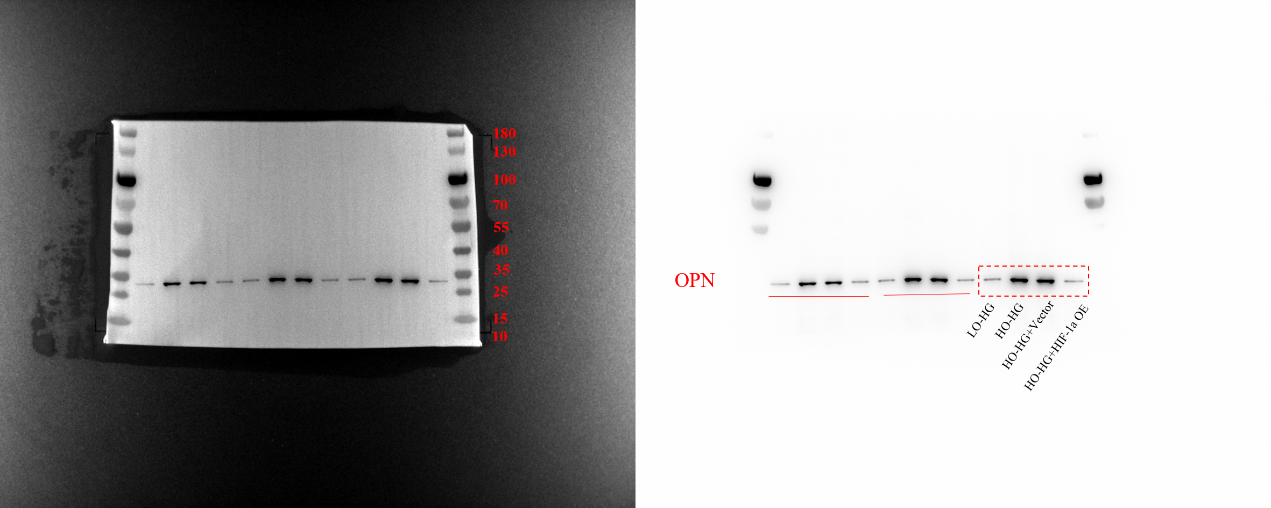

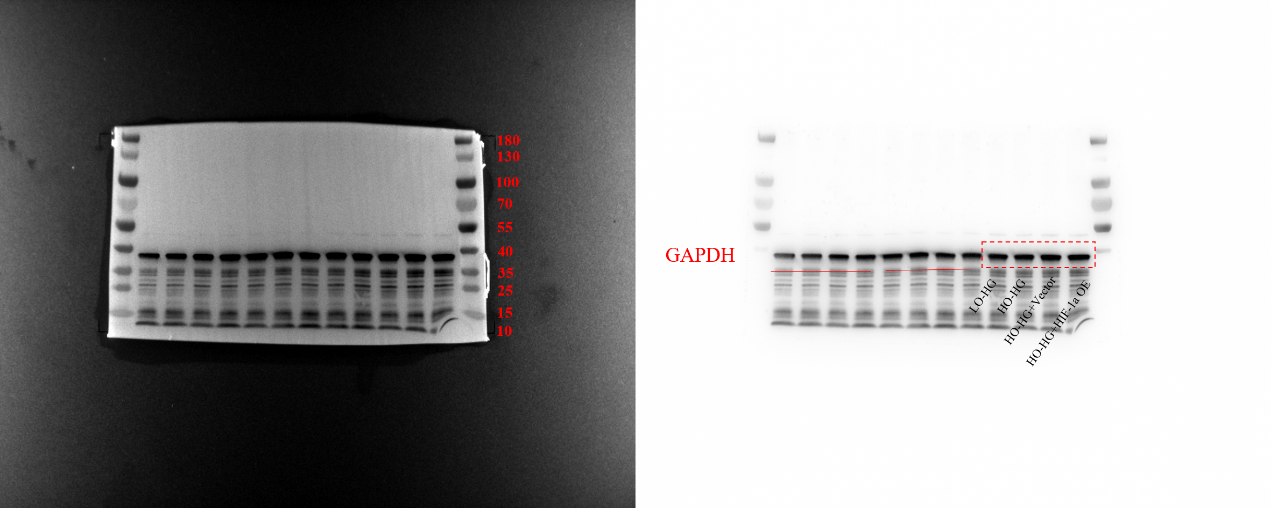
**

**Figure 6M.** Original western blot gels of RUNX-2 and OPN in the TSPCs. GAPDH was included as a loading control.

**
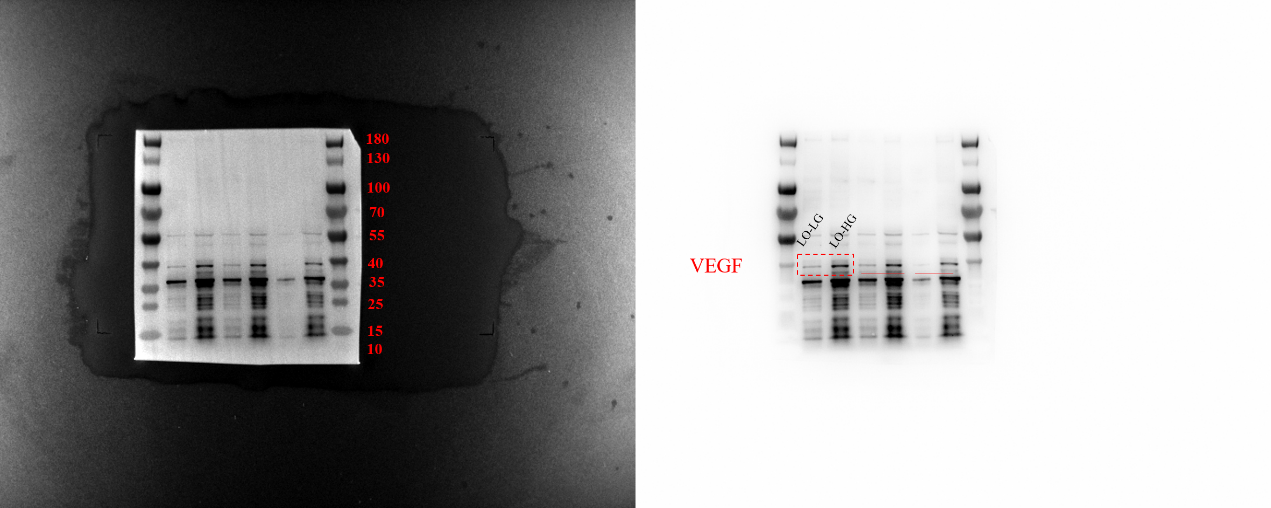
**

**
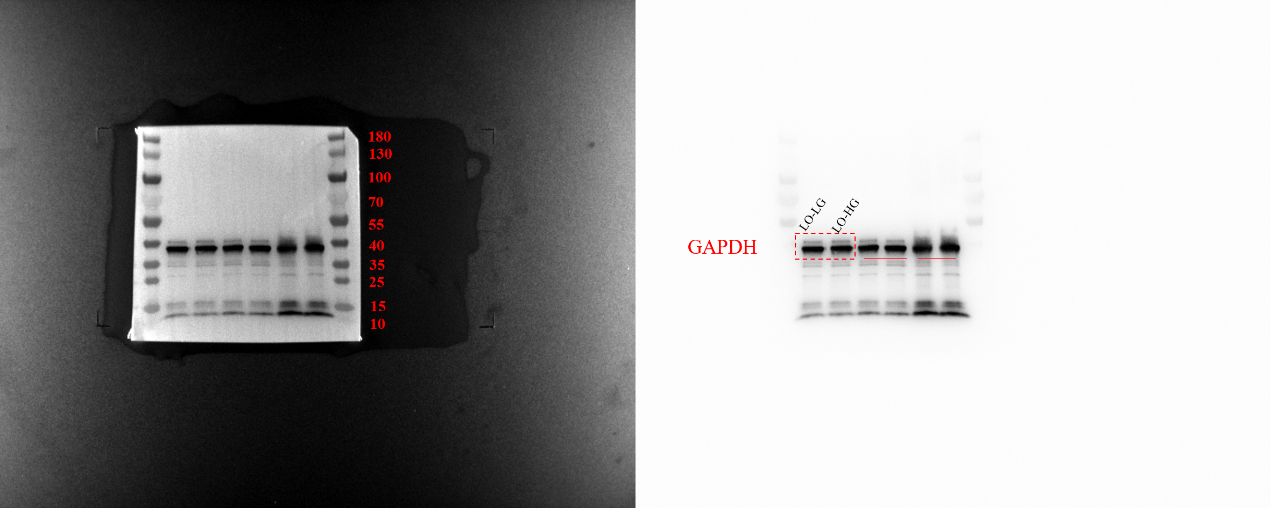
**

**Figure S2B.** Original western blot gels of VEGF in the TSPCs. GAPDH was included as a loading control.

**
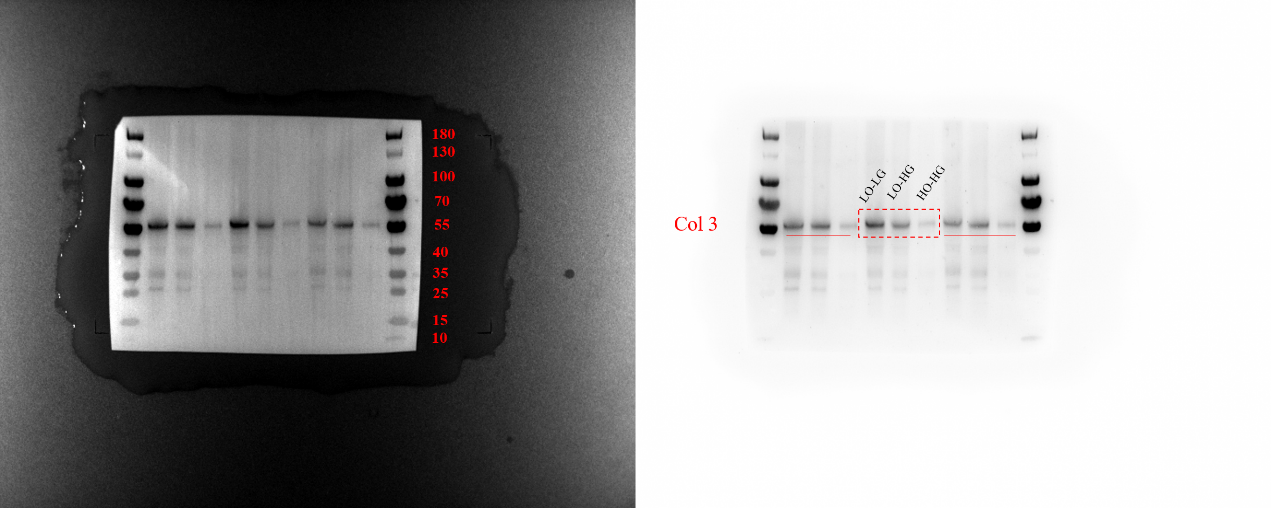

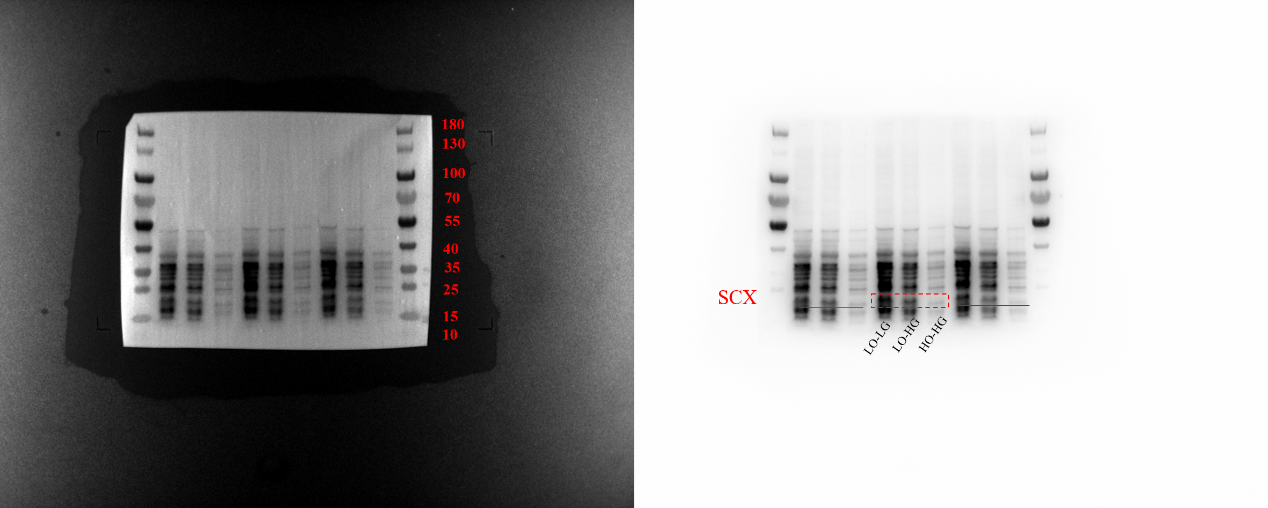

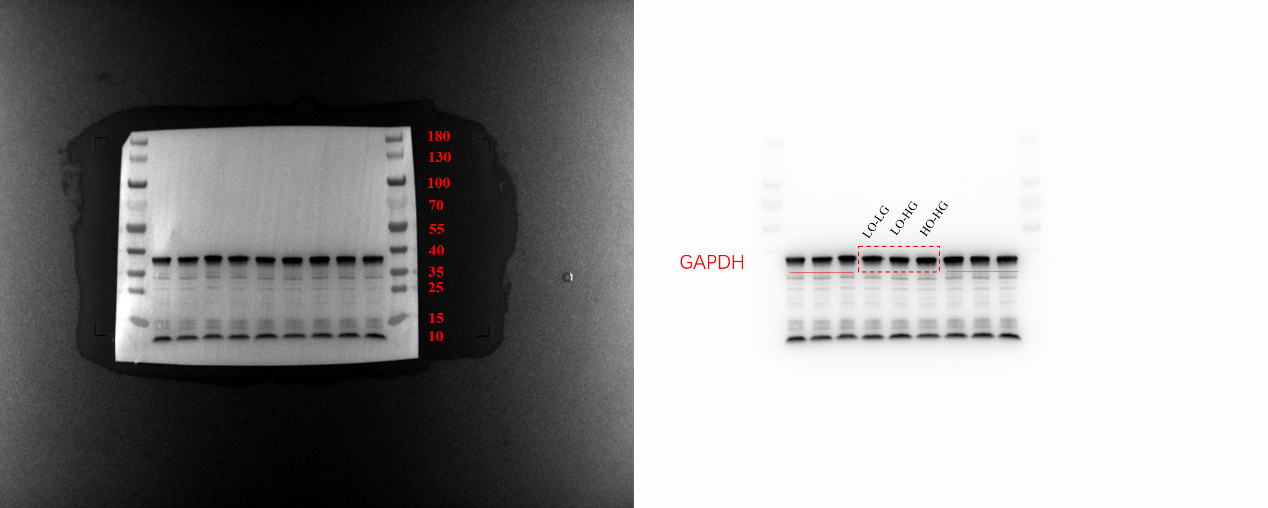
**

**Figure S4F.** Original western blot gels of Col 3 and SCX in the TSPCs. GAPDH was included as a loading control.

**
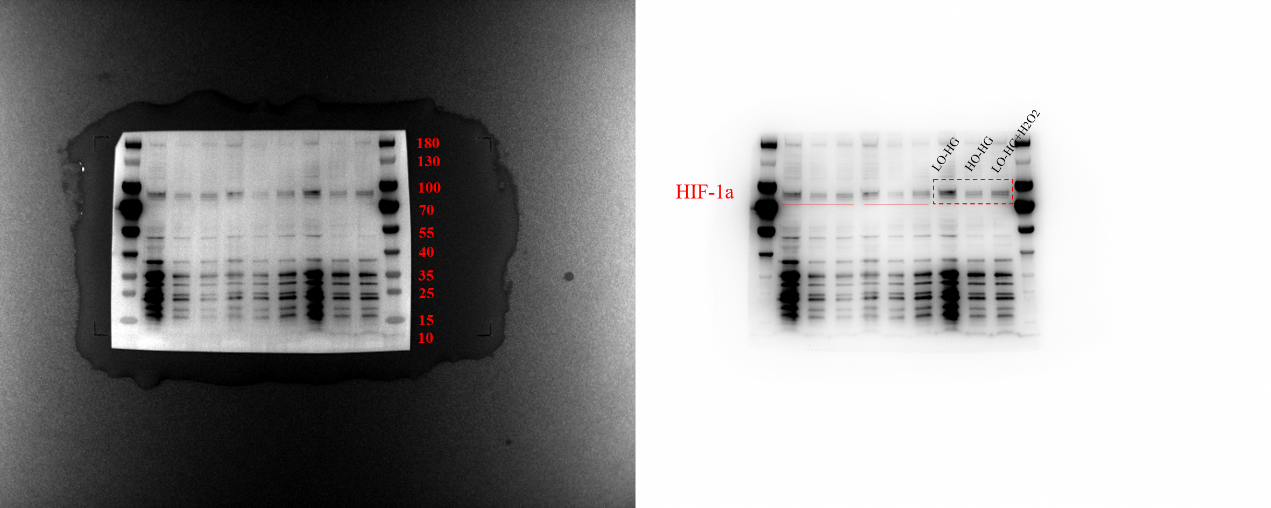

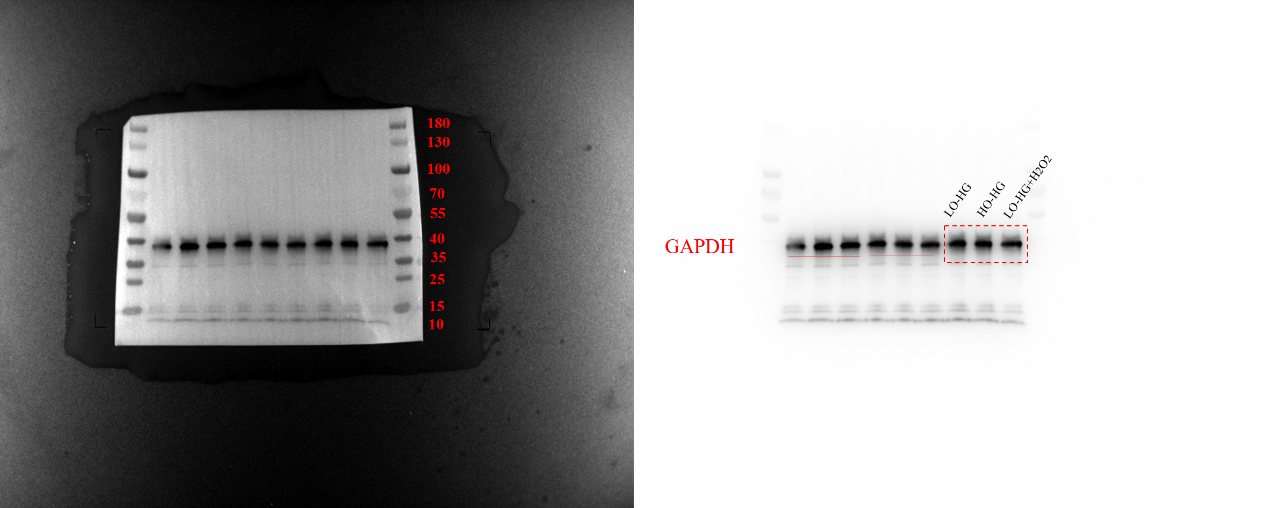
**

**Figure S6B.** Original western blot gels of HIF-1a in the TSPCs. GAPDH was included as a loading control.

**
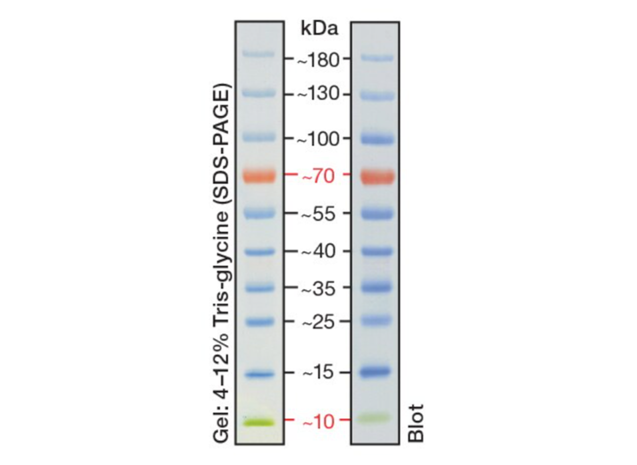
**

Protein Ladder（Thermo Fisher Scientific, MA, USA）
